# Critical dynamics in the spread of focal epileptic seizures: Network connectivity, neural excitability and phase transitions

**DOI:** 10.1371/journal.pone.0272902

**Published:** 2022-08-23

**Authors:** S. Amin Moosavi, Viktor K. Jirsa, Wilson Truccolo

**Affiliations:** 1 Department of Neuroscience, Brown University, Providence, RI, United States of America; 2 Aix Marseille University, INSERM, INS, Institut de Neurosciences de Système, Marseille, France; 3 Carney Institute for Brain Science, Brown University, Providence, RI, United States of America; Aristotle University of Thessaloniki, GREECE

## Abstract

Focal epileptic seizures can remain localized or, alternatively, spread across brain areas, often resulting in impairment of cognitive function and loss of consciousness. Understanding the factors that promote spread is important for developing better therapeutic approaches. Here, we show that: (1) seizure spread undergoes “critical” phase transitions in models (epileptor-networks) that capture the neural dynamics of spontaneous seizures while incorporating patient-specific brain network connectivity, axonal delays and identified epileptogenic zones (EZs). We define a collective variable for the spreading dynamics as the spread size, i.e. the number of areas or nodes in the network to which a seizure has spread. Global connectivity strength and excitability in the surrounding non-epileptic areas work as phase-transition control parameters for this collective variable. (2) Phase diagrams are predicted by stability analysis of the network dynamics. (3) In addition, the components of the Jacobian’s leading eigenvector, which tend to reflect the connectivity strength and path lengths from the EZ to surrounding areas, predict the temporal order of network-node recruitment into seizure. (4) However, stochastic fluctuations in spread size in a near-criticality region make predictability more challenging. Overall, our findings support the view that within-patient seizure-spread variability can be characterized by phase-transition dynamics under transient variations in network connectivity strength and excitability across brain areas. Furthermore, they point to the potential use and limitations of model-based prediction of seizure spread in closed-loop interventions for seizure control.

## Introduction

The nature and the size of seizure spread in a patient with focal epilepsy can vary substantially across different seizures. In many cases, a focal seizure may start and remain localized until its termination. These cases often lead to subclinical seizures, i.e. seizures that because of scant spread, do not generate observable clinical symptoms. Other seizures may spread to different extents. Seizure spread dynamics thus tend to cause functional impairments (e.g. loss of consciousness, susceptibility to sudden death, etc) across different seizures.

These variations in spread dynamics and its extent remain poorly understood. The problem is particularly relevant in the case of pharmacologically resistant focal seizures, where the two main therapeutic alternatives are neurosurgical resection of epileptogenic areas and open- or closed-loop seizure control. In the former, properties of seizure spread may guide the decision process for resective neurosurgery [1]. In the latter, a closed-loop approach (e.g. RNS System, NeuroPace) consists of detecting a focal seizure as early as possible and triggering electrical stimulation to prevent spread [2–4].

In this study, we focus on the phenomenological level rather than specific biophysical mechanisms. We examine seizure spread in the context of a recently proposed mathematical model of focal seizure dynamics [5–8]. The epileptor network model is typically formulated as a neural mass network model, where each network node representing a particular brain area is modeled by a system of ordinary differential equations. The model captures the spontaneous transition dynamics of going in and out of seizures. Transitions are controlled by a slow variable reflecting dynamics in ionic concentrations, metabolic factors, etc, which are slower than the neural dynamics of the seizure itself. Importantly, network connectivity is based on patient-specific connectivity among brain areas. This connectivity is derived from white-matter tractography imaging usually estimated via diffusion tensor MRI. In addition, for each patient, identified seizure onset areas or epileptogenic zones (EZs) are implemented in the network model as nodes with pathologically high excitability levels. Variations of the epileptor network model have been successfully applied to various problems such as the determination of target areas for neurosurgical resection to prevent seizures or control their propagation [9, 10], and to explain various properties of the neural dynamics in focal seizures [5, 6, 11, 12], including a taxonomy of focal seizure onset types [8]. Especially relevant to this study, initial applications of epileptor-networks, estimated from patient-specific structural connectivity and electrophysiological data, can predict the main qualitative features (e.g. recruited areas and the temporal ordering) of the actual seizure spread observed in those patients [13, 14].

Specifically, we work with the hypothesis that variations in seizure spread across different seizures in the same patient result in large part from transient fluctuations in dynamic neural excitability and global functional connectivity strength. These transient fluctuations can reflect different behavioral or physiological states (e.g. [15, 16]) and show many time scales ranging from minutes to hours and days (e.g. [17, 18]). We examine this hypothesis by performing simulations of patient-specific epileptor network models under different levels of global connectivity strength, excitability of non-EZ nodes (surrounding areas to seizure onset areas to where seizure might spread) and input noise variance.

Excitability levels for the surrounding nodes are always kept at a non-epileptic levels. In other words, the dynamical system consisting of only the surrounding nodes (without an active EZ) is always stable and non-epileptic. Furthermore, we focus on the scenario where the EZ nodes are known, and ask whether a seizure will spread given that it has just started in a particular EZ node. Among all the identified EZ nodes in a given patient, we assume that only one is the site of seizure onset at a particular time. The remaining identified EZ nodes remain at non-epileptic excitability levels. As indicated above, the potential target application would be a closed-loop system (e.g. RNS System, NeuroPace) where a focal seizure has been detected in its very beginning and the question is to determine if and how the seizure will spread in order to better guide spatio-temporal stimulation. We also consider the scenario where an EZ node undergoes a seizure when isolated from the network, and ask whether that will also hold in the case of this EZ node being connected to network. In this way, we address inhibitory and restraining effects of the surrounding not only on seizure spread itself, but also on seizure initiation.

In order to define seizure spread as a simple collective state of the epileptor networks, we ignore the details of the simulated temporal dynamics in each network node and thus work with a highly coarse-grained version of the dynamics. Specifically, after each simulation we assign to each node binary states: 1 if the node was recruited into a seizure and 0 otherwise. Seizure spread is then quantified simply as the spread size (number of nodes that have been recruited into the seizure, including the active EZ node) or the corresponding spread fraction. We also preserve information about onset and offset seizure activity in each node for later assessment of predictability of the temporal ordering of surrounding nodes recruitment into a seizure. We examined the phase diagrams of this collective variable as a function of excitability and global connectivity strength. We show that seizure spread dynamics behaves akin to critical phase transitions. In this study, we use the terms critical and criticality in a broader sense than in their corresponding use in statistical mechanics as specific to second-order phase transitions. Here, criticality relates primarily to loss of stability and bifurcations when examining qualitative transitions in the dynamics of stochastic neural networks [19, 20].

## Materials and methods

### Epileptor network model

The epileptor network model has been described in several previous studies (e.g. [6, 11]). For completeness we include here the equations following closely the notation in [10]. For an *N*-node patient-specific epileptor network model, the dynamics are given by

$$\begin{array}{c} \begin{array}{c} {\dot{x}}_{1,i}=y_{1,i}-f_{1}\left(x_{1,i},x_{2,i}\right)-z_{i}+I_{1} \end{array} \end{array}$$ (1)

$$\begin{array}{c} \begin{array}{c} {\dot{y}}_{1,i}=\frac{1}{\tau_{1}}\{1-5x_{1,i}^{2}-y_{1,i}\} \end{array} \end{array}$$ (2)

$$\begin{array}{c} \begin{array}{c} {\dot{z}}_{i}=\frac{1}{\tau_{0}}\{4\left(x_{1,i}-x_{0,i}\right)-z_{i}-w\sum_{j=1}^{N}W_{ij}\left[x_{1,j}\left(t-\tau_{ij}\right)-x_{1,i}\left(t\right)\right]\} \end{array} \end{array}$$ (3)

$$\begin{array}{c} \begin{array}{c} {\dot{x}}_{2,i}=-y_{2,i}+x_{2,i}-x_{2,i}^{3}+I_{2}+0.002\;g\left(x_{1,i}\right)-0.3\left(z_{i}-3.5\right)+\xi_{i}\left(t\right) \end{array} \end{array}$$ (4)

$$\begin{array}{c} \begin{array}{c} {\dot{y}}_{2,i}=\frac{1}{\tau_{2}}\left\{-y_{2,i}+f_{2}\left(x_{2,i}\right)\right\}+\eta_{i}\left(t\right) \end{array} \end{array}$$ (5)

where

$$\begin{array}{c} \begin{array}{c} g\left(x_{1,i}\right)=\int_{t_{0}}^{t}e^{-\gamma (t-s)}x_{1,i}\left(s\right)\;ds, \end{array} \end{array}$$ (6)

and

$$\begin{array}{cc} f_{1}\left(x_{1,i},x_{2,i},z_{i}\right) & =\{\begin{array}{cc} x_{1,i}^{3}-3x_{1,i}^{2} & \;\text{if}\;x_{1,i}<0\\ \left(x_{2,i}-0.6{\left(z_{i}-4\right)}^{2}\right)\;x_{1,i} & \;\text{if}\;x_{1,i}\geq 0 \end{array}\\ f_{2}\left(x_{2,i}\right) & =\{\begin{array}{cc} 0 & \;\text{if}\;x_{2,i}<-0.25\\ 6(x_{2,i}+0.25) & \;\text{if}\;x_{2,i}\geq -0.25, \end{array} \end{array}$$

for *i* = 1, 2, …, *N*. The terms *ξ*_*i*_(*t*) and *η*_*i*_(*t*) correspond to stochastic inputs. (We effectively interpret the above as stochastic differential equations in the Itô calculus sense; see Model simulations). We set the parameters *I*_1_ = 3.1, *I*_2_ = 0.45, *γ* = 0.01, *τ*_0_ = 6667, *τ*_1_ = 1, *τ*_2_ = 10.

The coupling weights *W*_*ij*_ are obtained from patient-specific connectivity matrices derived from white-matter tractography with corresponding axonal transmission delays *τ*_*ij*_ (see Structural network connectivity).

The parameter *x*_0,*i*_ denotes the neural excitability in the *i*^th^-node. An epileptogenic zone (EZ) can be instantiated by setting *x*_0,*i*_ = −1.6 in the corresponding node. This excitability level ensures that an isolated EZ node undergoes a seizure. We set *x*_0,*i*_ to non-epileptic values in the non-EZ nodes, specifically *x*_0_ ∈ [−2.3, −2.09] as the critical excitability for an isolated node is $x_{0}^{c}\approx -2.061$, i.e. a node *i* is considered epileptogenic (an EZ node) if $x_{0,i}>x_{0}^{c}$. The parameter *w* corresponds to the global connectivity strength.

Briefly, given the time scale separation *τ*_0_ ≫ *τ*_2_ ≫ *τ*_1_, slow and fast oscillations in typical epileptic focal seizures are captured by the systems (*x*_1,*i*_, *y*_1,*i*_) and (*x*_2,*i*_, *y*_2,*i*_), respectively, while the slower time-scale permittivity variable *z*_*i*_ might reflect the generic dynamics of changes in extracellular ionic concentrations (e.g. potassium), metabolic factors, etc. The diffusive coupling [*x*_1,*j*_(*t* − *τ*_*ij*_) − *x*_1,*i*_] incorporates the hypothesis that a seizure spreads by epileptic activity in an EZ, perturbing the ionic/metabolic homeostasis in a distal area. This perturbation then results in distal areas also going into seizure. For a detailed analysis of the epileptor dynamics see [7, 8, 21].

### Structural network connectivity, time delays and epileptogenic areas

Patient-specific connectivity networks were inferred from white-matter tractography obtained via diffusion MRI [6, 22]. In patient-specific networks P1—P2, a 84-area (Desikan-Killiany) parcellation of brain areas was used, while for P3-P5 a finer 162-area parcellation was employed.

The inferred patient-specific connectivity matrices **W** were further truncated and normalized. To attenuate potentially large artifacts in the estimation of white-matter connectivity, entries larger than the 95% percentile (across all entries) were set to this threshold value. After that, all entries were normalized by this threshold such that 0 ≤ *W*_*ij*_ ≤ 1. Connectivity matrices for patients P1—P5 are shown in S1 Fig in S1 File.

Time delays *τ*_*ij*_ in the model were computed from the length of the estimated white-matter fiber tracts connecting brain areas related to nodes *i* and *j* [22]. The maximum length in the 5 different patient-specific connectivity data corresponded to 200 mm. We set the speed to 60 mm per time unit in the model. Given our choice of setting the time unit in the model to 0.02 s (see Model simulations), a speed of 3000 mm / s (i.e. 60 mm × 1/0.02s) was obtained, with a maximum time delay of 0.067 s for the largest fiber length of 200 mm. This is slower than expected when compared to only axonal transmission delays, and it should be understood here as including also delays in synaptic activity and rising times in neuronal population responses, for example. The time delays for each patient-specific network are shown in S1 Fig in S1 File. We also explored a range of much slower delays, but found no qualitative differences in the spreading dynamics in these epileptor network models. We elaborate on related issues in the Discussion section.

The following nodes and brain areas were identified as the epileptogenic zones (EZs)

P1: {61, 64} for ctx-rh-lingual and ctx-rh-parahippocampal, respectively;
P2: {48, 60, 81} for right-amygdala, ctx-rh-lateral orbito frontal and ctx-rh-temporal pole, respectively;
P3: {135, 154, 156} for right-rhinal-cortex, right-hippocampus-anterior and right-amygdala, respectively;
P4: {35, 51, 53, 54, 73, 74, 75} for left-temporal-pole, left-collateral-sulcus, left-parahippocampal-cortex, left-rhinal-cortex, left-hippocampus-anterior, left-hippocampus-posterior, left-amygdala, respectively;
P5: {83, 87, 116, 126, 135, 154, 156} for right-orbito-frontal-cortex, right-F3-pars-opercularis, right-temporal-pole, right-T2-anterior, right-rhinal-cortex, right-hippocampus-anterior, right-amygdala, respectively.

### Model simulations

The epileptor network model (Eqs 1–5) were implemented as a system of stochastic differential equations in the Itô calculus sense, where *ξ*_*i*_(*t*) and *η*_*i*_(*t*) thus denote Wiener processes. For numerical integration we used the Heun’s method, with the Wiener process increments implemented as i.i.d. Gaussian random variables with zero mean and variance *σ*^2^ ⋅ Δ*t*. The above five-dimensional node system was implemented as a 6-dimensional system by rewriting Eq 6 as corresponding differential equation. We wrote Python code to implement the numerical simulations and used an integration step of 0.05. To match typical oscillatory properties of focal epileptical seizures and for plotting purposes, we assumed a time unit in the model to correspond to 0.02 s. Given the simulation step size of 0.05, this resulted in a sampling rate of 1000 simulation steps per second.

The epileptor network was initialized with an initial conditions near identified stable equilibrium point of the single node epileptor model. The excitability parameter was set to the same nonepileptic value for all nodes (including all EZ nodes). Simulations were run for 20,000 time steps to let transients decay and the network to settle into a steady-state. At that point, the excitability for the selected EZ node was set to *x*_0_ = −1.6. Then, a seizure may or may have not developed in this active EZ node after some varying time interval depending on initial conditions and parameters. After a seizure terminated, we enforced a postictal (refractory) period by uncoupling the corresponding node where the seizure terminated from the network. This postictal period was enforced to all nodes regardless if the node was an EZ node or not.

We take seizure spread as a collective variable (see below) and examine its behaviour by taking *x*_0,*i*_ in the non-EZ nodes and *w* as varying control parameters. In addition, we examined noise drive with different variances and the effect of the location of the selected active EZ node. We varied *x*_0,*i*_ in the non-EZ nodes in a non-epileptic range, as well as the global coupling strength and the noise variance (e.g. Fig 2). We applied a grid to the parameter space (*x*_0,*i*_, *w*). For each point in the grid, we generated 30 stochastic realizations. The same approach was then applied to the case where a different node was selected as the active EZ node.

### Epileptor network observations, seizure spread as an order parameter, and critical dynamics

**Fig 1:**
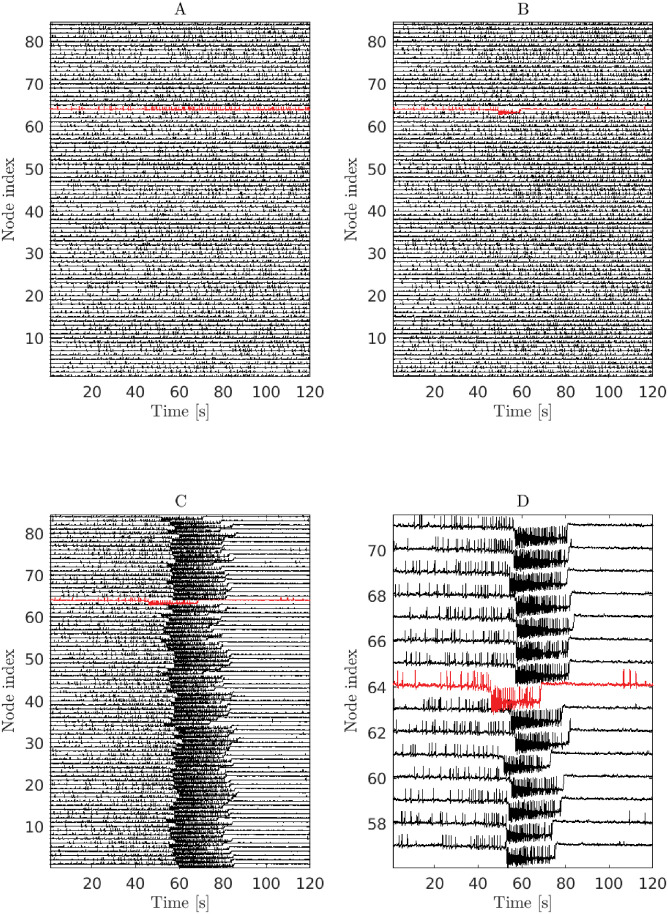
Seizure spread in the epileptor-network model. Three qualitatively different behaviors are observed for different global connectivity strength (*w*) and surrounding-node excitability (*x*_0_) parameter values. Figures show simulations of the epileptor network model for patient P1. Node 64 was identified as the target active EZ node (*x*_0_ = −1.6). The standard deviation of the noise input level was set to *σ* = 0.05. **(A)** The active EZ node (in red) was “inhibited” by the surrounding nodes in the network and, as a consequence, did not transition into a seizure. Global connectivity strength and excitability in the surrounding nodes were set to *w* = 1.6 and *x*_0_ = −2.3, respectively. **(B)** The seizure started in the target EZ node, but there was no spread in the network (*w* = 0.9, *x*_0_ = −2.2). **(C)** The seizure started in the EZ node near time 50 s and spread to all of the network nodes (*w* = 0.9, *x*_0_ = −2.1). Spread onset times varied across different nodes. Partial spread to a subset of nodes can also occur (see Figs 2 and 8). **D** A zoomed in view of the activity shown in panel **C**. After a seizure terminated in any given node, the node went into a postictal refractory state implemented by uncoupling the node from the network and, in the case of the EZ node, the excitability parameter *x*_0_ was switched back to the same non-epileptic level of the surround. Interictal spikes are observed prior to the seizure onset. The decrease in the spike rate after the seizure terminates is due to both a “refractory” effect of the intrinsic node dynamics and to the enforced uncoupling.

For plotting and visualization purposes (e.g. Fig 1), each node in the full 6-dimensional epileptor node is observed via the difference of the variables *x*_2,*i*_ and *x*_1,*i*_, which are related to the slow and fast ictal oscillations, respectively.

In order to quantify seizure spread and define a corresponding collective state variable, we ignored the details of the temporal dynamics in each node of the simulated epileptor network, and considered only discrete states, i.e. whether a seizure had spread or not to a given node, together with the temporal ordering of the spread, i.e. which nodes spread first and so on. As seizure activity showed large amplitude deviations from normal activity, specific thresholds were easily identified to ensure accurate detection of seizure onset and termination. A seizure was detected to spread to a given node when the activity in the corresponding state variable *g* (Eq 6 rewritten as a differential equation) crossed a specified threshold. We chose the variable *g*, instead of *x*_1,*i*_ or *z*_*i*_, because it presented a smoother activity.

Thus, the state of each node was then represented as a binary variable (1 for seizure and 0 otherwise). The collective state variable or order parameter was then defined as the spread size (number of nodes that entered seizure activity) across the network in any given simulation.

Given simulations of epileptor networks and the observed behavior of the defined order parameter, we built empirical phase diagrams as a function of control parameters, here excitability in the surround nodes and global connectivity strength. Qualitative changes in the order parameter, e.g. spread vs no spread, were used to construct phase diagrams and phase transition curves, i.e. curves tat separate distinct phases.

We refer to the region near these phase transition curves as near-criticality regions. As stated in the Introduction, here we use the terms critical and criticality in a broader sense than in the strict use in statistical mechanics. Briefly, in that context, critical point and criticality refer strictly to second-order phase transitions [23, 24]. In second-order equilibrium phase transitions, the order parameter (in many cases given by the 1st-order derivative of the free energy with respect to an external field) changes continuously as control parameters are varied around a critical point (i.e. there are no jumps in the phase transition), while response functions (second-order derivatives of the free energy wrt control parameters; e.g. magnetic susceptibility in Ising models) and correlation length diverge as power-law functions at the critical point in the thermodynamic limit *N* → ∞. In nonequilibrium systems [24], critical phenomena has mostly been characterized as absorbing state phase transitions [25], self-organized criticality [26], and branching processes [27] where the distribution of event (avalanche) sizes and durations in the system appear as power-law scale-invariant functions. Here, in the case of seizure spread in epileptor network models, the exact nature of the nonequilibrium phase transitions in the defined order parameter (spread size) remains an open question. For this reason, we use the terms critical and criticality in a broader sense where they relate primarily to bifurcations in stochastic dynamical systems. In this case, a critical point refers to a specific value of a control parameter where a bifurcation happens, regardless of whether the order parameter changes continuously or jumps, and whether response functions diverge [19, 20].

### Local linear stability analysis

Fixed points for the epileptor network model were computed by solving for the zeros of the deterministic system of differential equations (Eqs 1–6) with the Matlab function fsolve (Mathworks, Inc). We first determined the fixed points for a single-node epileptor. Given our choice of parameters and initial conditions for the numerical search, we found three fixed points: one typically stable, and the other two unstable. When solving for fixed points of the epileptor network, we started with initial conditions near to the stable fixed point found for the single-node epileptor model.

For local linear stability analyses, we write the Jacobian matrix for the 6-dimensional N-node epileptor network model as:

$$J=\left[\begin{array}{cccccc} J_{x_{1},x_{1}} & J_{x_{1},y_{1}} & J_{x_{1},z} & J_{x_{1},x_{2}} & J_{x_{1},y_{2}} & J_{x_{1},g}\\ J_{y_{1},x_{1}} & J_{y_{1},y_{1}} & J_{y_{1},z} & J_{y_{1},x_{2}} & J_{y_{1},y_{2}} & J_{y_{1},g}\\ J_{z,x_{1}} & J_{z,y_{1}} & J_{z,z} & J_{z,x_{2}} & J_{z,y_{2}} & J_{z,g}\\ J_{x_{2},x_{1}} & J_{x_{2},y_{1}} & J_{x_{2},z} & J_{x_{2},x_{2}} & J_{x_{2},y_{2}} & J_{x_{2},g}\\ J_{y_{2},x_{1}} & J_{y_{2},y_{1}} & J_{y_{2},z} & J_{y_{2},x_{2}} & J_{y_{2},y_{2}} & J_{y_{2},g}\\ J_{g,x_{1}} & J_{g,y_{1}} & J_{g,z} & J_{g,x_{2}} & J_{g,y_{2}} & J_{g,g} \end{array}\right],$$ (7)

where each component is itself an *N* × *N* matrix. The non-zero components are $J_{x_{1},x_{1}}=-\frac{\partial f_{1}}{\partial x_{1}}$, $J_{x_{1},x_{2}}=-\frac{\partial f_{1}}{\partial x_{2}}$, $J_{x_{1},z}=-\frac{\partial f_{1}}{\partial z}-I$, $J_{x_{1},y_{1}}=I$, $J_{y_{1},y_{1}}=-I$, $J_{y_{1},x_{1}}=-5\frac{\partial (x_{1}^{2})}{\partial x_{1}}$, $J_{z,z}=-\frac{1}{\tau_{0}}I$, $J_{x_{2},y_{2}}=-I$, $J_{x_{2},z}=-0.3I$, $J_{x_{2},g}=0.002I$, $J_{x_{2},x_{2}}=\frac{\partial (x_{2}-x_{2}^{3})}{\partial x_{2}}$, $J_{y_{2},y_{2}}=-\frac{1}{\tau_{2}}I$, $J_{y_{2},x_{2}}=-\frac{1}{\tau_{2}}\frac{\partial f_{2}}{\partial x_{2}}$, $J_{g,x_{1}}=I$, **J**_**g**,**g**_ = −*γ* **I**, and $J_{z,x_{1}}$ is given by

$$\begin{array}{c} J_{z_{i},x_{1,j}}=\frac{1}{\tau_{0}}\{\delta_{i,j}\left(4+w\sum_{k}W_{ik}\right)-\left(1-\delta_{i,j}\right)wW_{ij}\}. \end{array}$$ (8)

In the above, **I** is the *N* × *N* identity matrix, $\frac{\partial f}{\partial x}$ is a diagonal matrix with the *i*^th^ diagonal element equal to $\frac{\partial f(x_{i},\ldots )}{\partial x_{i}}$), and *δ*_*i*,*j*_ is the Kronecker delta function.

As explained in the Results section, we consider two instantiations of the above Jacobian matrix. In the first case, the EZ node is included in the analysis and the Jacobian remains a 6*N* × 6*N* matrix. In this case, we set *x*_0,*i*_ = −1.6 for the EZ node, and a non-epileptic *x*_0_ value homogeneously for all of the remaining nodes, and proceed with the linear stability analysis.

In the second case, the EZ node is replaced by an “external” constant input to its target surrounding nodes and the Jacobian is a 6(*N* − 1) × 6(*N* − 1) matrix obtained by removing the rows and columns related to the EZ node. The active EZ node contribution to the nodes in this network was replaced by a constant input ${\bar{x}}_{1,EZ}$ computed by averaging *x*_*EZ*,1_ during the seizure period.

This constant input to the nonepileptic nodes was mediated via the same diffusive couplings via the permittivity variable *z* as in the usual epileptor network. For example, assuming the *i*^th^-node as the active EZ node, its contributions to the variable *z*_*j*_ in the *j*th nonepileptic node was given by $w\times W_{ji}\;\left[x_{1,j}\left(t-\tau_{ij}\right)-{\bar{x}}_{1,EZ}\right]$. We note that this constant input does not appear in the Jacobian matrix itself, but it changes the fixed point at which the Jacobian is evaluated (The fixed points of the 6(*N* − 1) × 6(*N* − 1) system). The constant input shifts the fixed points of the system towards instability.

The combined use of the eigenvalues of both Jacobians lead to the best prediction of the phase diagrams for seizure spread in the epileptor networks. We also note that time delays were ignored, i.e. we set *τ*_*ij*_ = 0 in the derivation of the Jacobian matrices. Proix et al. (2014) have argued that because the interaction between nodes is via the slow permittivity variable *z*, axonal propagation delays play no major role on bifurcations of equilibrium points and thus on the seizure spread dynamics. Each Jacobian matrix was evaluated at the fixed point of the corresponding epileptor network system.

We also considered local stability analysis and related predictions based on a 2-dimensional reduction of the epileptor model given by [6, 9]

$$\begin{array}{c} \begin{array}{c} {\dot{x}}_{i}=-x_{i}^{3}-2x_{i}^{2}+1-z_{i}+I_{1}+\xi_{i}\left(t\right) \end{array} \end{array}$$ (9)

$$\begin{array}{c} \begin{array}{c} {\dot{z}}_{i}=\frac{1}{\tau_{0}}\{4\left(x_{i}-x_{0,i}\right)-z_{i}-w\sum_{j=1}^{N}W_{ij}\left[x_{j}\left(t-\tau_{ij}\right)-x_{i}\left(t\right)\right]\}, \end{array} \end{array}$$ (10)

where *τ*_0_ = 6667 and *I*_1_ = 3.1.

### Prediction of the temporal order of node-recruitment during seizure spread

When a seizure spread is predicted, i.e. the real part of the maximal eigenvalue of the Jacobian matrix satisfies *λ*_*R*_ > 0, we used the corresponding eigenvector to predict the order of spread or node-recruitment during the seizure. Specifically, we computed the magnitude of the leading eigenvector components corresponding to the variable *z*_*i*_, for nodes *i* = 1, 2, …, *N* in the surrounding, i.e. excluding the active EZ node. Nodes with larger component magnitudes were predicted to be recruited earlier during the seizure than nodes with smaller component magnitudes.

## Results

### Epileptor network model: Seizure simulations

Focal epileptic seizures were simulated with the epileptor network model. The intrinsic dynamics in each network node is formulated as a 6-dimensional ODE system, which captures the generation of interictal spikes, slow and fast oscillations during the seizure itself, and a much slower process (permittivity variable) that spontaneously brings the network in and out of seizures (Fig 1; Methods).

The networks were instantiated with 5 different patient-specific (inter-node) connectivity matrices, axonal propagation time-delays, and corresponding identified epileptogenic zones (Methods). In two of the cases (P1 and P2), connectivity matrices were obtained with an 84-node parcellation of the brain areas, and 162-node parcellation in the remaining (P3-P5). Corresponding connectivity weight matrices together with descriptive graph theoretic measures are given in the S1 Fig and S1 Table in S1 File. A list of the EZ nodes and corresponding brain area labels is given in the Methods section. While the connectivity matrices are symmetric and non-negative, diffusive coupling via the permittivity variables in different nodes allows not only for excitatory but also inhibitory and restraint effects in the network (Fig 1; Methods).

Each seizure was simulated by starting the entire network in a non-epileptic excitability level (*x*_0_ parameter in the model; Methods). After a specified time to allow network dynamics transients to settle down, the excitability level for a single selected target EZ node was set to an epileptic level (*x*_0_ = −1.6). This target EZ node may or may have not then transitioned into seizure after varying times, and led to partial or full seizure spread, depending on the level of surrounding node excitability (homogeneous *x*_0_ across nodes), global connectivity strength (parameter *w*), and on the different stochastic realizations for a given input noise level (Fig 1). We simulated thousands of stochastic realizations under variations of the above parameters and EZ node selections.

### Seizure spreading dynamics behave akin to phase transitions

**Fig 2:**
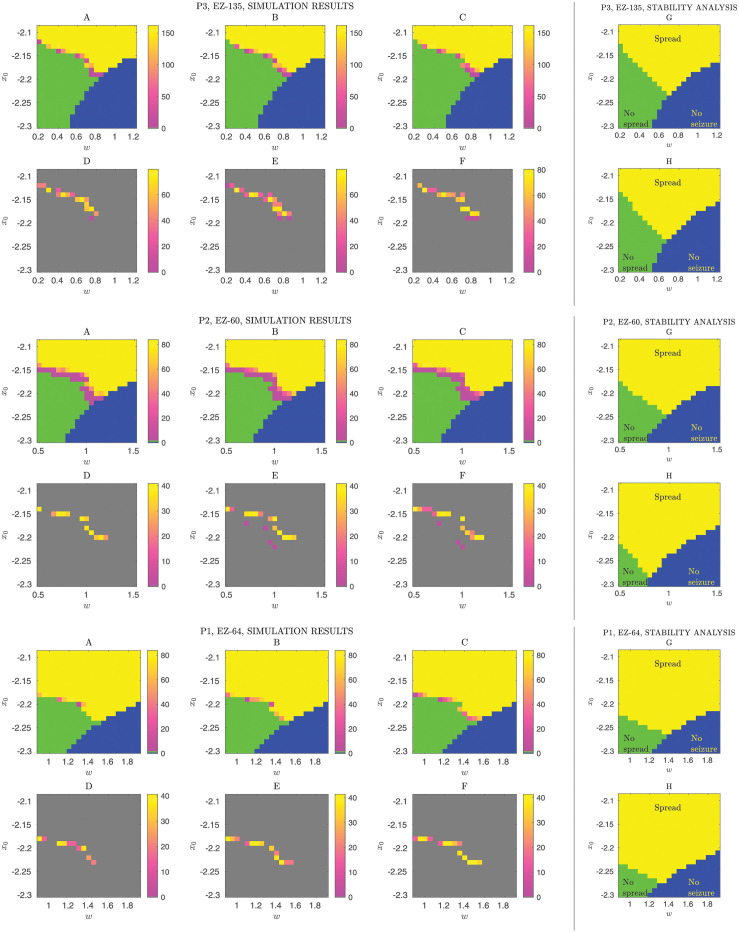
Seizure-spread phase diagrams. Seizure spread is examined as a collective state variable, i.e. spread size or number of seizing nodes, under variations of global connectivity strength (*w*) and homogeneous surrounding node excitability (*x*_0_). **Top (A,B,C)** The phase diagrams were obtained by simulating the epileptor network (patient P3, node 135 corresponds to the active EZ, with *x*_0_ = −1.6) for three different noise strengths (*σ* = 0.05, *σ* = 0.10, *σ* = 0.15). Blue, green and yellow colors represent no seizure, no spread and seizure spread, respectively, obtained from 30 realizations of the stochastic dynamics for a given parameter setting (each point in the diagram). The colorbar indicates spread size. **(D,E,F)** Fluctuations across stochastic realizations are observed in the near-criticality region between the no-spread and spread phases as indicated by the standard deviation of the spread size (30 different realizations). The gray background corresponds to regions of no observed fluctuations. **(G)** Phase diagram predicted by the local stability analysis applied to the deterministic 2D-reduced epileptor network model (Methods). **(H)** Phase diagram predicted by the analysis of the deterministic original epileptor network model. Prediction of the different phases was based on the leading eigenvalues of two different Jacobian matrices, one that included the active EZ node and another that replaced the EZ node with a constant input (see Methods and Fig 3 for a schematic description). Although the true location and shape of the transition curves differ, the predictions capture well the qualitative aspects of the phase diagram. **Middle (A-H)** The panels show another example from patient P1 with node 64 as the active EZ. **Bottom (A-H)** Example from patient P2 with node 60 as the active EZ, showing distinct fluctuation regions in contrast to the above examples.

As stated above, we defined seizure spread as a collective variable given by the spread size (or equivalently fraction of seizing nodes in the network) for any given patient-specific epileptor network simulation. To characterize qualitative different states or phases of this collective variable, we computed phase diagrams as a function of control parameters given by the surround excitability, global connectivity strength, noise levels and location of the active or target EZ node. Phase diagram examples for three different patient-specific networks, active EZ node, and parameter settings are shown in Fig 2. (See S1 File for more examples from all 5 patients and the respective active EZ nodes).

Three main phases with clear phase transition curves appear: (a) no spread, i.e. the seizure remains localized in the target EZ node and does not spread to the surrounding network nodes; (b) the seizure spread across the network with various different spread sizes; in particular, partial spread to a subset of network nodes is observed near a small near-criticality region surrounding the phase transition curve; (c) no seizure, i.e. a seizure does not occur even in the active EZ area, in other words, the surround network activity appears to inhibit seizure onset in the target EZ area.

The existence of these three phases and their qualitative features were general across all patient-specific networks, and across different active EZ areas in the same patient (S2-S8 Figs in S1 File). These findings emphasize the role of excitability in the surrounding network and of global connectivity strength, as well as the role of inhibitory and restraint effects enabled by diffusive coupling, on seizure spread. Our results extend the 2-node epileptor network analysis in [6] to patient-specific networks with much larger number of nodes and under broader parameter and noise level variation.

### Phase diagrams of seizure spread show large stochastic fluctuations in a narrow near-criticality region

By running simulations with different random noise realizations and noise levels, but fixed excitability and global connectivity strength and target EZ node, we examined the existence of stochastic fluctuations in the defined collective variable for seizure spread. Additive noise in the epileptor network model reflects the combined effect of various types of neural noise, including thermal noise in ion channels, failure in neurotransmitter vesicle release in synapses, and many other unaccounted factors.

The existence of stochastic fluctuations in the collective variable for the same levels of surround excitability and global connectivity strength has important practical implications, for instance in the predictability of seizure spread. Large stochastic fluctuations of the collective variable across different seizures under the same parameter setting make predictability harder even when one has a good estimate of surrounding neural excitability levels and global connectivity strength.

We observed stochastic fluctuations only near the phase transition curve separating the no spread and spread phases (Fig 2D–2F). Fluctuations were confined to the small near-criticality region surrounding this curve. Away from this region, seizure spread dynamics appeared deterministic for the examined noise levels and number of stochastic realizations. Similar results were obtained for other patient-specific networks (S2-S8 Figs in S1 File). In a small number of cases, unconnected fluctuation regions appeared. They consisted of two side-by-side regions with a no-fluctuation zone in between (e.g. Fig 2D–2F, bottom panel, P2, node 60 as the active EZ; S4 Fig in S1 File: P3, node 154 as the active EZ). This phenomenon can be understood as follows. In those cases, a couple of nodes were strongly connected to the active EZ node. As the the parameter variation moves toward traversing the phase transition boundary, say by increasing both {*w*, *x*_0_}, initially only those strongly connected surrounding nodes go into seizure and with fluctuations across realizations. Then, with a further slight increase, these nodes always go into seizure, so there are no fluctuations. With a further increase, the seizure starts to spread more broadly and with fluctuations.

The nature of the fluctuations in this near-criticality region may have important implications for prediction and control of seizure spread. In particular, if focal seizure dynamics in actual brains follow similar dependencies on excitability and global connectivity strength, we expect prediction of seizure spread to be an easy task when the brain is away from this near-criticality region, but challenging otherwise.

### Local linear stability analysis predicts the phase diagrams and the temporal order for node-recruitment during seizure spread

We examined whether local linear stability analysis of epileptor network models could predict the phase diagrams obtained from their simulations (Methods). In addition to the epileptor network models, we considered also a 2-dimensional reduced model version [6].

Fig 2G and 2H shows examples for three patient-specific network connectivity and a specific target EZ node. See S2-S8 Figs in S1 File for all the other target EZ nodes and patient-specific networks. Overall, despite differences in the exact locations of the phase transition curves, these predictions captured very well the qualitative structure of the phase diagrams based both on the full-dimensional (6D) model and an its 2D reduced version. Nevertheless, the local linear stability analysis appeared to overestimates the spread region in the phase diagrams, in particular near transition curve between no-spread and spread. This difference between simulation-based results and linear stability analysis prediction may result in part from the finite number (30 in this case) of stochastic realizations. At these two phase boundaries, the probability of spread events can be very small such that a very large number of realizations might be needed to observe such events.

**Fig 3:**
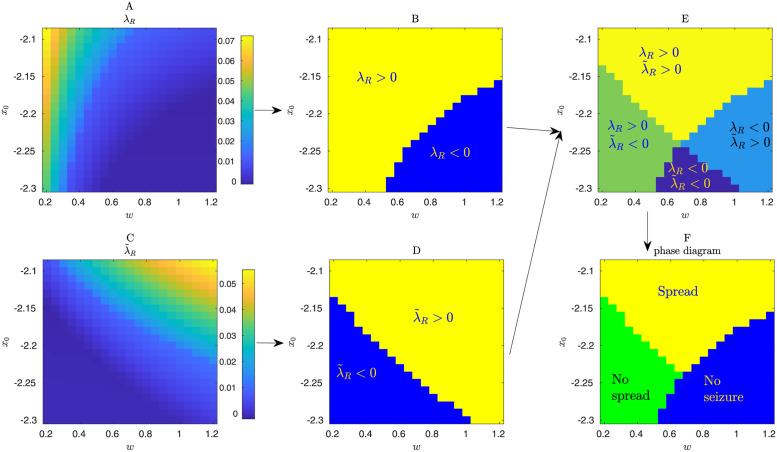
Schematics for the linear stability analysis of the deterministic epileptor network. Prediction of phase diagrams is based on the leading eigenvalues of two different Jacobian matrices evaluated at a given fixed point, and for a given global connectivity strength (*w*), homogeneous surrounding-node excitability (*x*_0_), patient-specific connectivity and given active EZ node. **(A,B)** In this case, the Jacobian matrix is derived from a network that includes the active EZ node. The term λ_*R*_ denotes the real part of the corresponding largest eigenvalue of this Jacobian matrix. The fixed point of the system is stable for λ_*R*_ < 0, implying no seizure will happen, and unstable (seizure) otherwise. Based on this analysis alone one cannot predict the phase diagram for the seizure spread. **(C,D)** In this case, the Jacobian matrix (and corresponding leading eigenvalue $\tilde{\lambda }$), are derived from an epileptor network where the active EZ node has been removed and replaced by a constant external input to nodes connected to it. This external input is related to the average seizure activity in the active EZ node (Methods). This analysis alone also cannot predict the phase diagram, in particular the phase where seizure in the active EZ node is “inhibited” by the surrounding nodes. **(E,F)** The joint consideration of the signs of the leading eigenvalues of the two Jacobian matrices leads to qualitatively correct phase diagrams for seizure spread as illustrated in Fig 2. Diagrams corresponds to patient P3 and node 135 as the active EZ area (*x*_0_ = −1.6). Time delays are ignored in this linear stability analysis (Methods).

Briefly, these phase diagram predictions were obtained as follows. We considered two different Jacobian matrices, i.e. local linearizations around a given equilibrium point of the epileptor network dynamics. One of the Jacobian matrices was derived from the network that included the active target EZ node, while the other was derived from a network where this active EZ node was replaced by a constant input to its connected surrounding nodes (see Fig 3 and Methods for details). Based on the leading eigenvalues of these two matrices, we predicted the corresponding phase of collective variable. We predicted seizure spread when the two leading eigenvalues indicated an unstable fixed point, i.e. when Re(λ) > 0 for both of the leading eigenvalues of the two matrices. (We note that while the patient-specific structural connectivity matrix is symmetric, the Jacobian matrices are not since they include also asymmetric block diagonals that reflect the intrinsic node dynamics.) The other phases of the collective variable were predicted by different combinations of the sign of the real part of these two eigenvalues (Fig 3).

**Fig 4:**
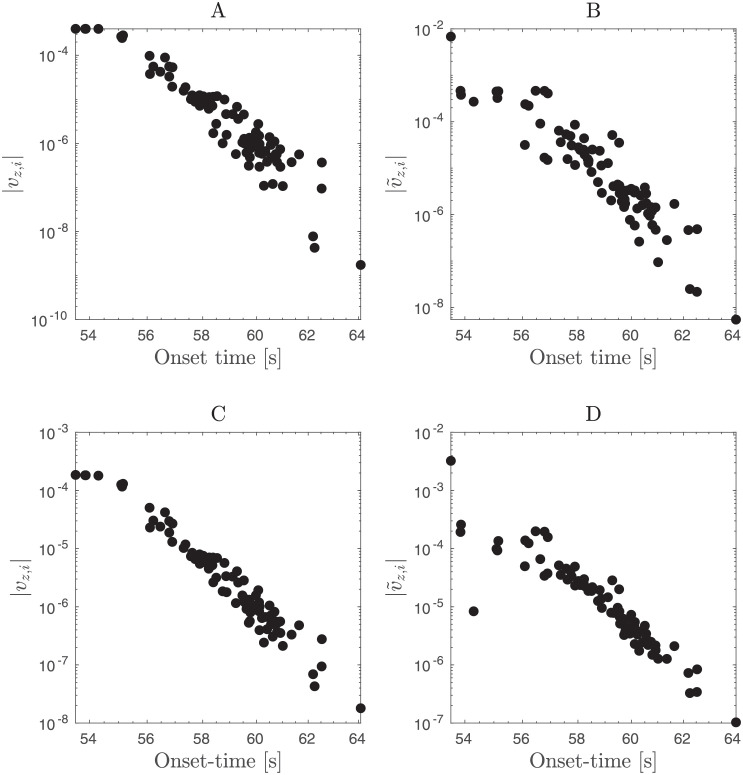
Relationship between the leading eigenvector of the Jacobian matrix and the temporal order of seizure spread (seizure onset times) across different nodes. The magnitude of the components of the leading eigenvector of the Jacobian matrix from the epileptor network are denoted by |*v*_*z*,*i*_|, with *z*, *i* indicating that the component relates to the permittivity variable *z* in the *i*th node. Similarly for $|{\tilde{v}}_{z,i}|$, but now for the leading eigenvector computed from the Jacobian matrix where the active EZ node is replaced by a constant input to nodes connected to it (see Fig 3). **(A,B)** panels are based on the 2D-reduced epileptor network model while **(C,D)** panels are based on the original epileptor network model. Note that these magnitudes predict roughly wells the temporal order of seizure spread across the network, in particular the magnitudes based on the Jacobian matrix of the network that includes the active EZ node. The examples were obtained from a single stochastic realization for patient P1, node 64 as the active EZ (*x*_0_ = −1.6), surrounding-node excitability *x*_0_ = −2.13, global connectivity strength *w* = 1.1, and noise level at *σ* = 0.05. See Fig 5 for a statistical summary for different patient-specific connectivity matrices, different active EZ locations, noise levels and stochastic realizations.

**Fig 5:**
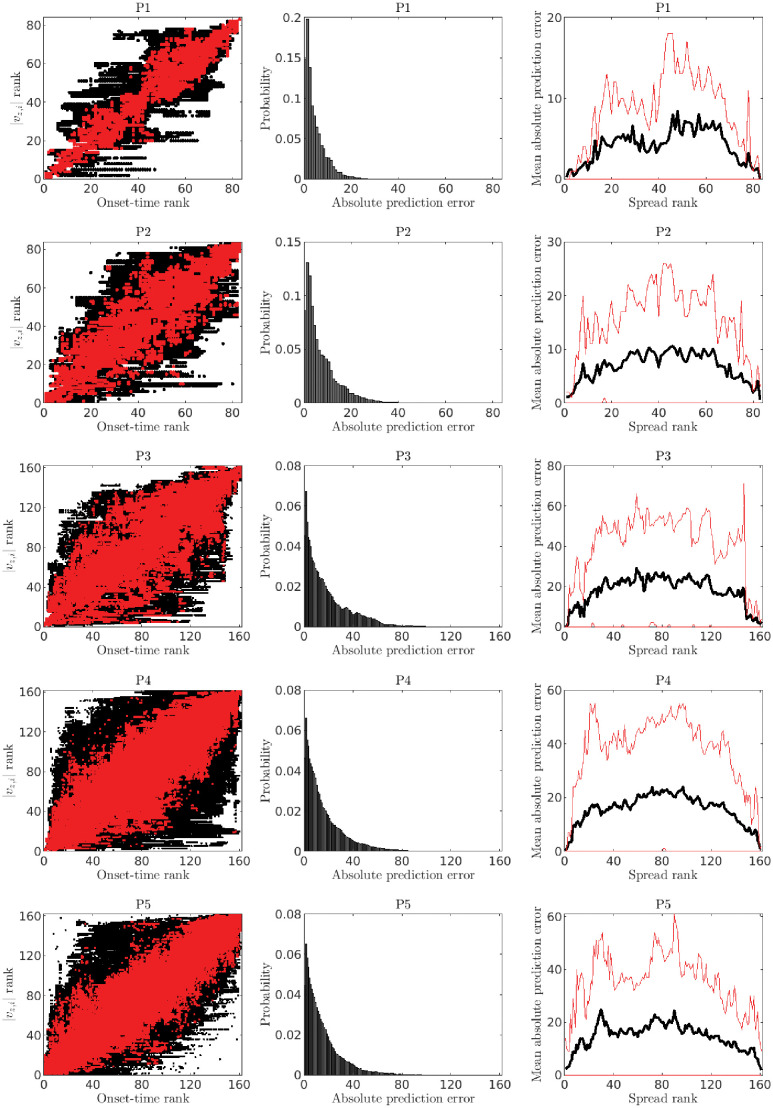
Prediction of temporal order of seizure spread based on linear stability analysis: Statistical summary. Each row corresponds to a different patient-specific connectivity (P1—P5 from top to bottom). **Left column**: the panels show the onset-time rank (rank 1 corresponds to a node which was first recruited into the seizure, and so on) and the corresponding seizure spread rank predicted by the ranked magnitudes of the leading eigenvector of the Jacobian matrix for a given epileptor network. As before, *v*_*z*,*i*_ indicates the eigenvector component related to the permitivity variable *z* in the *i*th node. Each black dot corresponds to a network with a particular active EZ node, global connectivity strength and surrounding-node excitability {*w*, *x*_0_} parameter set, noise level and stochastic realization. Three noise levels were examined (*σ* = 0.05, *σ* = 0.1, and *σ* = 0.15). The range of (*w*, *x*_0_) values were set such that seizures spread to the full network (e.g. within the yellow region in Fig 2). Thirty stochastic realizations were simulated for each parameter set, noise level and active EZ node. In each case, *x*_0_ = −1.6 for the active EZ node. The overlapping red dots correspond to the empirical 95% confidence regions. As seen for all patient-specific connectivity matrices, true and predicted ranks tend to concentrate around the diagonal line. **Middle column**: the panels show the empirical probability distribution for the (integer) absolute error in the prediction of rank of recruitment during seizure spread. **Right column**: the panels show the mean absolute error of rank prediction (black) and the corresponding 95% confidence region (red). Prediction errors are very small for the nodes that are recruited first and last during a seizure, with the error increasing toward nodes that are recruited in between. For reference, a uniform random prediction of recruitment rank would show a mean prediction with a “U” shape, i.e. the opposite of these results.

Beyond predicting the phase diagrams for different patient-specific networks, we also assessed how well linear stability analysis predicted the temporal order in which each node in the network is recruited into seizure during the seizure spread. For that purpose, when the stability analysis indicated seizure spread (unstable fixed point), we ranked the magnitude of the (complex) components of the leading eigenvector (dominant mode) of the Jacobian matrix. (More specifically, we ranked the magnitudes for the eigenvector component related to the permittivity variable *z* in different nodes |*v*_*z*,*i*_|; see Methods.) The components of the leading eigenvector indicate the directions (nodes) along which the unstable dynamics diverges. Furthermore, nodes with larger corresponding magnitudes should be recruited first. These predictions agree well with the temporal order observed in the simulations as shown in Fig 4 for a specific example and in Fig 5 for a statistical summary across patient-specific connectivity matrices and all variations in global connectivity strength, surrounding node excitability, target EZ node, and noise levels.

**Fig 6:**
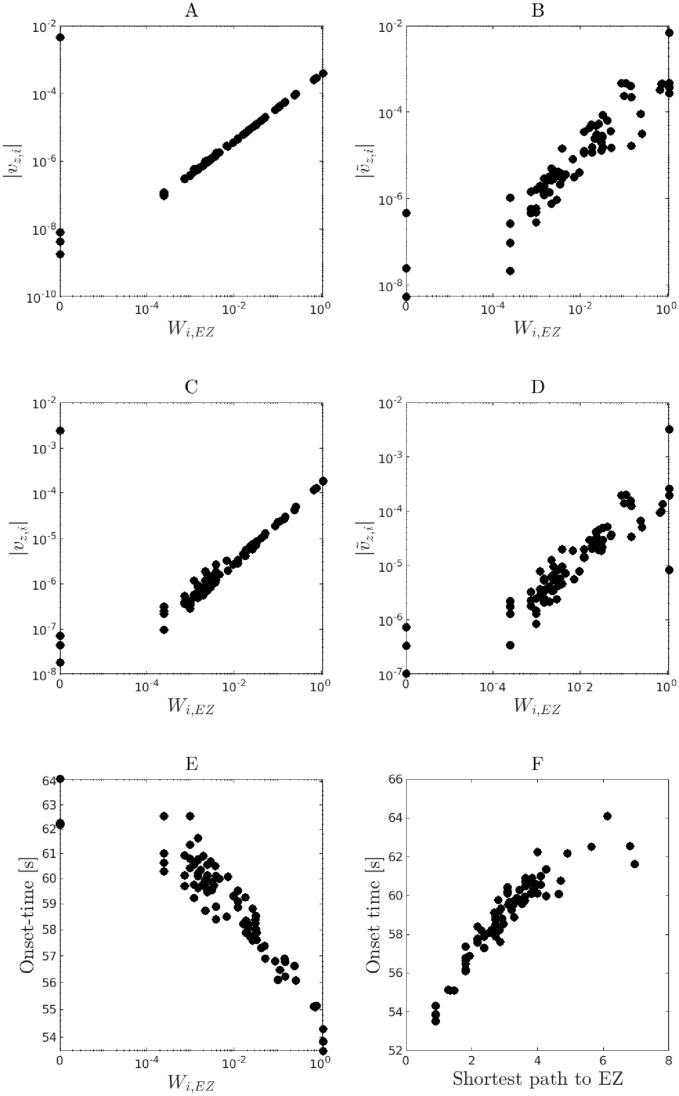
Relationship between the magnitude of the leading eigenvector components and the patient-specific connectivity matrix. The magnitudes of the leading eigenvector components |*v*_*z*,*i*_| are plotted versus the interaction weight *W*_*i*,*EZ*_ from the EZ area to the corresponding *i*th surrounding node. As before, *v*_*z*,*i*_ indicates the eigenvector component related to the permitivity variable *z* in the *i*th node. **(A,B)** panels are based on the 2D-reduced epileptor network model with the active EZ node included (**A**) or replaced by a constant input (**B**). **(C,D)** Similarly, but for the original epileptor network model. **E** Relationship between the seizure onset times for each network nodes and the corresponding interaction weight (*W*_*i*,*EZ*_) from a given active EZ node. **F** Similarly, but with respect to the shortest path between a given surrounding node and the corresponding active EZ node. The panels show an example from a single stochastic realization from patient-specific network P1, with node 64 as the active EZ (*x*_0_ = −1.6), surrounding node excitability set to *x*_0_ = −2.13, global strength connectivity *w* = 1.1, and noise level with *σ* = 0.05. The panels show strong relationships among the leading eigenvector components, the patient-specific connectivity, seizure recruitment times, and shortest path length from the active EZ node to surrounding nodes.

**Fig 7:**
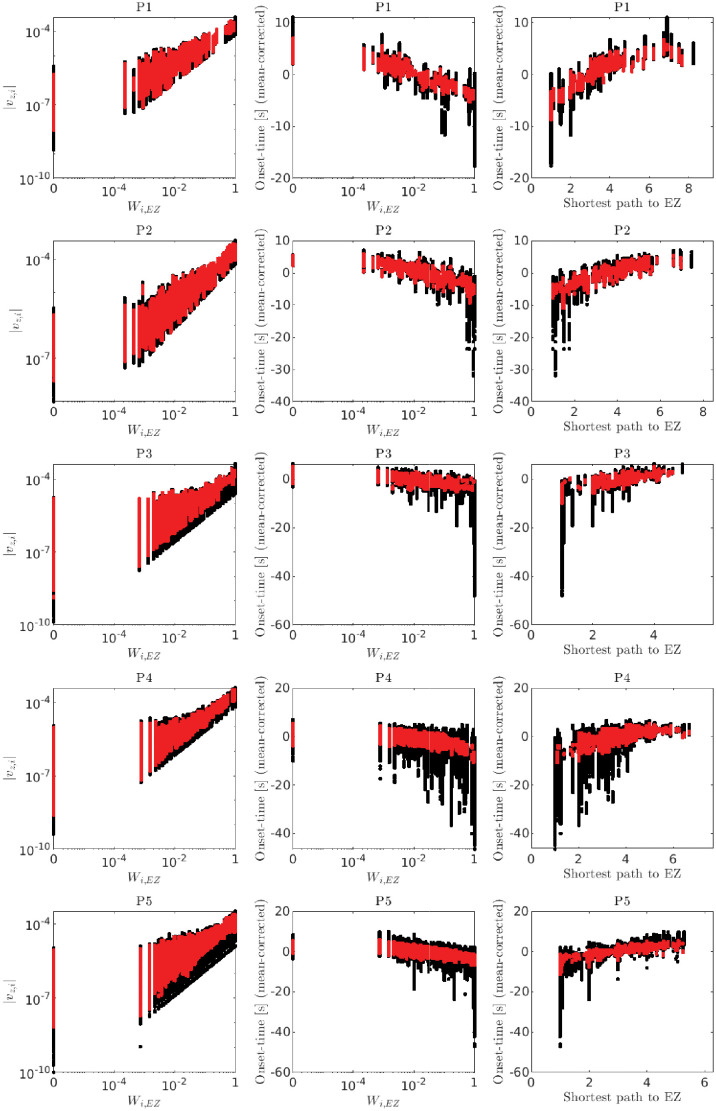
Relationships among magnitudes of leading eigenvector components, patient-specific connectivity matrices, seizure onset times and path lengths: Statistical summary. Same conventions as in Fig 6. Each row corresponds to a different patient-specific connectivity (P1, P2 and so on, from top to bottom). As in Fig 5, each dot corresponds to a network with a particular active EZ node, global connectivity strength and surrounding node excitability {*w*, *x*_0_} parameter set, noise level and stochastic realization. Three noise levels were examined (*σ* = 0.05, *σ* = 0.1, and *σ* = 0.15). The range of (*w*, *x*_0_) values were set such that seizures spread to the full network (e.g. yellow region in Fig 2). Thirty stochastic realizations were simulated for each parameter set, noise level and active EZ node. In each case, *x*_0_ = −1.6 for the active EZ node. Because different values of (*w*, *x*_0_) shift the seizure onset times in the target EZ node and corresponding spread onset times in the surrounding nodes, in each realization we corrected for these shifts by subtracting the averaged onset times over all nodes in that realization from onset times of each node that gives mean-corrected onset times.

Further analysis revealed a roughly linear (in logarithmic scale) relationship between the magnitudes of the components of the leading eigenvector and the connectivity weights from the activate EZ node to the surrounding nodes (Fig 6A–6D). Therefore, while the prediction of the phase of the collective variable requires examination of the eigenvalues of the Jacobian matrices, prediction of the temporal ordering and spread onset times for different surrounding nodes can also be easily read out from the patient-specific connectivity matrices (Fig 6E). We also examined the relationship between spread onset time and different graph theoretical measures computed from the patient-specific connectivity matrices, including the shortest path length between the surrounding nodes and the active EZ node in a given seizure, as well as corresponding betweeness centrality and clustering coefficients. Of those, only the shortest path length showed a strong linear relationship (Fig 6F). A statistical summary across patient-specific connectivity matrices, active EZ locations, (E,w) parameter variations, and stochastic realizations is given in Fig 7. The 95% confidence regions, shown in red color, indicate that the good statistical linear relationships, observed in Fig 6 for a specific case, are overall preserved.

The above analysis of predictability of spread rank across nodes during full spread brings the question of how much variability there is in the spread path across different stochastic realizations of the same epileptor network under fixed parameters. We found that, typically, ranks changed by only 1 or 2 across different realizations Thus, overall, there was little variability in the actual spread path across different realizations for the noise levels used here. (The choice of input noise variance levels was constrained in this study by the requirement that the surrounding nodes do not go spontaneously into seizure states. In other words, the seizure always starts at the seizure onset zone.). S9 and S10 Figs in S1 File shows this analysis in detail for two specific examples.

Finally, we examined the power of eigenvector centrality, rather than the leading eigenvector of the Jacobian matrix, to predict spread rank across nodes. Eigenvector centrality has been used in previous studies to predict different seizure stages and propagation (e.g. [28, 29]). In Network Science, the eigenvector centrality, computed on estimated adjacency matrices, is commonly used as a complementary quantity to examine network properties. One way to estimate these adjacency matrices is to first compute a functional connectivity matrix, e.g. a matrix estimated via pairwise crosscorrelation or spectral coherence functions. Here, we assessed this approach by first estimating functional connectivity based on extrema of cross-correlation functions computed from the simulated epileptor network time series according to [30]. Statistically significant pairwise correlations contributed to edges to the derived (binary, symmetric) adjacency matrices. For this particular case of epileptor networks, we observed no successful prediction based on eigenvector centrality. Furthermore, eigenvector centrality, computed directly from the actual structural (white-matter) patient-specific connectivity matrices, lead to similar results. (This is understandable since the structural matrix does not specify by itself the location of the epileptogenic node.) S11 and S12 Figs in S1 File show the analysis and additional details for two example cases. We emphasize that these findings seem specific to epileptor network models and explored parameter regions. As stated above, the usefulness of eigenvector centrality analysis has been demonstrated before in the case of recorded electric potentials during seizures.

### Seizure spread fluctuations in near-criticality regions show bimodal distribution

**Fig 8:**
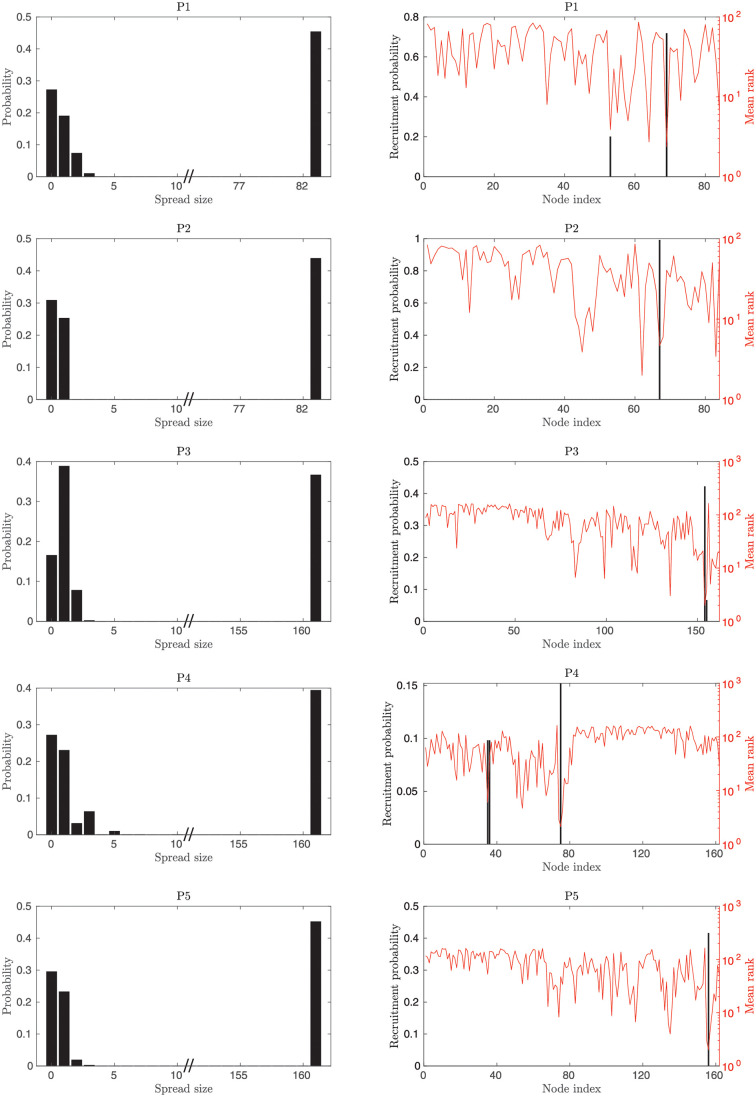
Relationship between statistical fluctuations in the near-criticality region and the recruitment rank for surrounding nodes based on linear stability analysis. **Left column** We examined the spread size (number of recruited surrounding nodes into the seizure; first column) when the epileptor network dynamics is in the near-criticality region (see Fig 2). Each row corresponds to a given patient-specific connectivity matrix. The empirical probability distributions show two clear modes of seizure spread in this region, one at small or no spread and the other at full network spread. These distributions are based on all of the {*w*, *x*_0_} parameter set choices that resulted in fluctuations in the phase diagrams, different active EZ nodes, three noise levels (*σ* = 0.05, *σ* = 0.1, and *σ* = 0.15) and 30 stochastic realizations for each case. **Right column** When a small spread happens, for a given active EZ node the same small set of nodes tends to be recruited across different seizures. We checked whether the recruited nodes and their recruitment probability corresponded to high predicted ranks (e.g. rank 1, 2, etc) based on the ranked magnitudes of the leading eigenvector components computed from the Jacobian matrix (with the active EZ node included in the network). For example, the panel A-2 shows that the node with highest probability of recruitment (black bar) in the small spread cases also had the highest mean rank (rank close to 1, red curve). Results are plotted for one of the active EZ nodes of patients that are from top to bottom P1:61, P2:60, P3:154, P4:51, P5:154. These results show that, conditioned on knowing whether one is dealing with a small or full spread, predictability based on linear stability analysis features is still possible in the near-criticality fluctuation region, although noisier than in the full spread phase. In the high fluctuation region, linear stability analysis will fail to predict the extent of spread, but if there is spread it can predict the temporal order of seizure recruitment.

We examined in more detail the nature of the collective variable fluctuations in the near-criticality regions, i.e. the narrow region surrounding the transition curve between the no-spread and seizure spread phases (Fig 2). Seizure spread size showed a bimodal distribution, with modes located at the two extremes: no or small partial spread and full spread across the network (Fig 8A).

We note that the absence of events in the intermediate spread size range is likely to reflect in part the range of explored noise levels as well as the number of stochastic realizations generated for each parameter set. In other words, the absence of events in intermediate spread size values might reflect their very low but finite probability, which could increase with higher noise levels. In any case, these results indicate a strong bimodal structure.

Finally, we verified that nodes from the same small subsets were recruited during small partial seizure spread in different realizations. Furthermore, the magnitudes of the leading eigenvector components |*v*_*z*,*i*_| corresponding to these nodes tended to be ranked much higher than the magnitudes of those components corresponding to nodes that were never recruited in the small partial seizure spread.

These results indicate that, conditioned on knowing whether one is dealing with a small partial seizure spread or a full spread event, linear stability analysis related quantities and network connectivity still provide some predictability in these near-criticality regions. A remaining problem for future examination is whether information in preictal activity in patient-specific epileptor network models can help improve the prediction of the seizure spreading dynamics, especially near-criticallity regions where stochastic fluctuations appear to lead to substantial variability in the extent of seizure spread across the surrounding areas.

## Discussion

We have shown that seizure spread, defined as a coarse discrete-state collective variable in patient-specific epileptor network models of focal epilepsy, behaves akin to phase transitions under control parameters including neural excitability in the network nodes surrounding an active epileptogenic area, the location of this area in the network, global connectivity strength and noise levels. Furthermore, the qualitative features of the phase diagrams, as well as the temporal order of node recruitment into seizure, were well predicted by measures derived from linear stability analysis at equilibrium points of the neural dynamics in the epileptor networks.

The two closest previous works to our study are [9, 31]. Proix et al. (2014, Fig 5) examined phase diagrams, but only for a generic 2-node epileptor network. In their work, phase diagrams were computed directly from numerical simulations, not from predictions based on local stability analyses. Additionally, due to the lack of spatial degrees of freedom, the effects of stochastic fluctuations across different realizations and control parameter settings were not possible to examine. Proix et al. (2017) showed that epileptor network models can be used to predict the propagation zone of seizures observed in 15 patients. However, no further analysis of the network models in terms of the prediction of the temporal order of seizure recruitment and spreading dynamics (phase diagrams) as a function of excitability and global coupling strength was performed. Furthermore, neither the variability of spread sizes and paths across different seizures (stochastic realizations) or the robustness of prediction based on linear stability analysis to stochastic fluctuations were systematically examined.

In contrast to the above two cited studies, in this manuscript we addressed the prediction of complete phase diagrams using connectivity matrices with much larger number of nodes (84 and 162) obtained from different patient-specific connectivity matrices. Furthermore, we also examined the role of stochastic fluctuations on the properties of the seizure spreading dynamics in epileptor networks, their effect on the variability of spreading dynamics across different stochastic realizations, and the nature of the fluctuations near criticality, i.e. near the phase transition curves. By looking at many stochastic realizations, we can also provide a better assessment of the predictability of seizure spreading based on local linear stability analyses in these network models. We think that the theoretical investigation of spreading dynamics in this class of models is an important first step towards their application to actual seizure spread prediction in the context that motivates this study, i.e. closed-loop seizure spread prediction and control as done in applications such as the NeuroPace RNS System [4, 18], for example.

Our specification of time conduction delays between different areas or nodes in the epileptor networks is a rough conservative approximation of node-to-node interaction delays in the context of the modeled macroscopic neural dynamics, incorporating not only axonal conduction delays but also synaptic and neural population responses. As stated in the Methods section, delays due exclusively to myelinated (white-matter) axonal transmission between different brain areas can be much smaller. Studies in human and non-human primates show comparable delay times to those used in this manuscript. Studies in monkeys [32] involving cortico-cortical (white-matter) LIP-FEF axons with a distance of ∼ 30 mm show conduction times in the range of 0.5 ms to 8 ms, while cortico-thalamic V1-LGN axons show conduction times in the range of 2 ms to 20 ms. Intracranial electrical stimulation studies in the human brain have identified single-pulse early CCEP responses within < 100 ms (common to both healthy and epileptogenic areas) and later delayed responses (related to epileptiform induced discharges) occurring in the range of 100 ms—600 ms [33]. Similarly, Keller et al. [34] show cortico-cortical evoked potentials (ECoG recording/stimulation) with early phase (absolute amplitude A1) responses within < 50 ms, with (Euclidean) distances from the stimulation site varying from ∼ 20 mm to 160 mm. More recently, Trebaul et al. [35] have systematically examined CCEP onset and peak latencies, and their relation to fiber lengths, using a probabilistic tractography approach. Ranges of onset latencies varied from 20 ms to ∼ 50 ms for corresponding distances of 10–20 mm to 90–100 mm.

Interaction time delays have been shown to play a crucial role in both neural dynamics and synaptic plasticity [36–39]. However, as stated earlier, although we have also explored a range of time delays (including much longer delays than mentioned above), we have not observed major effects in the qualitative features of the spreading dynamics in these epileptor network models. That seems to be the case because, as stated earlier, the time-scale of these delays (∼ 70 ms for the two most distant nodes—connected by ∼ 200-mm-long white-matter fibers in the models) are still much faster than the time scale of the actual spreading dynamics in the epileptor networks.

Our analysis of the nature of the phase transitions, in particular the transitions across the no spread and spread phases, is limited by the relatively small size of examined patient-specific epileptor network models and the number of stochastic realizations. We hope in the future to address how the properties of these phase transitions scale as the network size increases. The development of an explicit probabilistic model will also help towards this goal. A related issue is the distribution of seizure spread sizes. Our findings show bimodal distributions (with modes concentratated near small and full spread, respectively) in these relatively small epileptor network models. We are not aware of human brain recordings during seizures in the same patient, especially over a reasonable number of area and seizures, to allow the assessment of actual distributions of spread sizes. As an example of currently available data based on SEEG recordings of focal seizures, all cases in the 15-patient dataset in [31] show partial spread confined to a few areas. Cases of secondary generalization would require a much broader SEEG coverage for spread assessment than commonly done. Explosive/threshold spreading dynamics, which would lead to corresponding bimodal distributions with modes near small and almost full spread, have been suggested in previous theoretical studies involving for example networks of Kuramoto oscillators (e.g. [40]. We conjecture that a more continuous range of spread size (in particular power law distributions) might be possible in this class of models. That would be enabled by critical points (second-order phase transitions) in the excitability and global connectivity strength parameter space. We hope to examine this issue with more abstract probabilistic models of seizure spreading dynamics.

Another important issue not examined here is that of path dependent phase transitions. Here, we considered only the scenario where one knows a focal seizure has just started and is still very localized to a seizure onset area. (That is also the main application context we envision here, e.g NeuroPace RNS System devices to prevent seizure spread.) Then, the question is: Given knowledge of white-matter connectivity, excitability (*E*), global connectivity strength (*w*), will the seizure spread or not, and if it does spread, how? We are considering the network dynamics in a very small time window (the spread prediction window) just after seizure onset where both *E* and *w* are roughly steady. Therefore, we are in a very specific point in the derived phase transitions diagrams. There, for a specific (*E*, *w*), we can then predict which phase (spread, partial spread, no spread) the network will present. We are not examining the problem of how the phase of the order parameter changes with changes in *E* and *w*. That would depend on many things including the rates of change *dE*/*dt* and *dw*/*dt*, initial conditions and the actual paths in the control parameter (*E*, *w*) space. The resulting phase diagrams could be path dependent. There could be hysteresis effects, etc. We hope to address the issue of path-dependent phase transitions in future studies.

We hope to contrast the linear stability based measures used here to other approaches related to synchronizability [41] and constraints on the coupling weights themselves derived from stability master functions [42], network node-fragility [43, 44] and control theoretic analysis [45, 46]. Two other remaining problems are the examination of how inhomogeneous neural excitability and deviations from symmetry in patient-specific structural connectivity matrices affect the phase diagrams and the overall dynamics for seizure spread. Nevertheless, regarding symmetric node-to-node connectivity, it is worth mentioning that Jirsa et al. [11] and Proix et al. [31] show that a symmetric white-matter connectivity can still capture the directed spreading dynamics observed in actual human seizures. Despite symmetric white-matter connectivity in the network models, directed interactions and spread also depend, as shown here, on the path length with respect to the seizure onset area, diffusive coupling, excitability parameters, etc, which can all be adjusted to different patient cases.

Our study was restricted to model simulations due in large part to the lack of extensive electrophysiological recordings in people with focal epileptic seizures. Most available data comes from recordings obtained in epilepsy monitoring units prior to resective neurosurgery. These typically include only a few seizures per patient, relatively small covering of brain areas, and potential confounding factors introduced by post-anesthesia and surgical effects. Thus, accurate data about the actual nature and size of seizure spread to which contrast model predictions on a patient by patient manner remains limited. We expect the future development of new brain recording technologies including also the measurement of neural excitability and related parameters during long time periods under daily life conditions will address these limitations.

Patient-specific epileptor network models and related adaptations can be fitted directly to intracranial EEG recordings [8, 13, 14] and be used for prediction of seizure spread and characterization of seizure onset taxonomies. Although much larger and systematic studies are required to assess how epileptor-network models generalize to different epilepsy types and etiologies, these previous studies have involved up to 88 patients, including cortical dysplasia, tumors, mesial temporal sclerosis. Our results indicate that predictability of seizure spread should be fairly simple for brains operating outside near-criticality regions, but challenging within these regions where large stochastic fluctuations tend to be present. That is expected to be the case even when accurate estimates of network connectivity, excitability levels in surrounding nodes and global connectivity strength are available. We think that the complementary use of predictive information obtained from preictal activity and nonlinear measures, together with the linear stability analysis examined here, are two potential directions towards improving predictability in those cases.

## Supporting information

S1 File (PDF)
