# Supplementary material for: Critical dynamics in the spread of focal epileptic seizures: Network connectivity, neural excitability and phase transitions
Source: PLoS One. 2022 Aug 23;17(8):e0272902. doi: 10.1371/journal.pone.0272902 (PMC9397939; doi:10.1371/journal.pone.0272902)
Supplement: S1 File — (PDF) [file pone.0272902.s001.pdf]

Critical dynamics in the spread of focal epileptic seizures: network connectivity, neural excitability and phase transitions

Supplementary Information

S. Amin Moosavi, Viktor K. Jirsa and Wilson Truccolo

Epileptor network model: patient-specific structural connectivity matrices and time delays

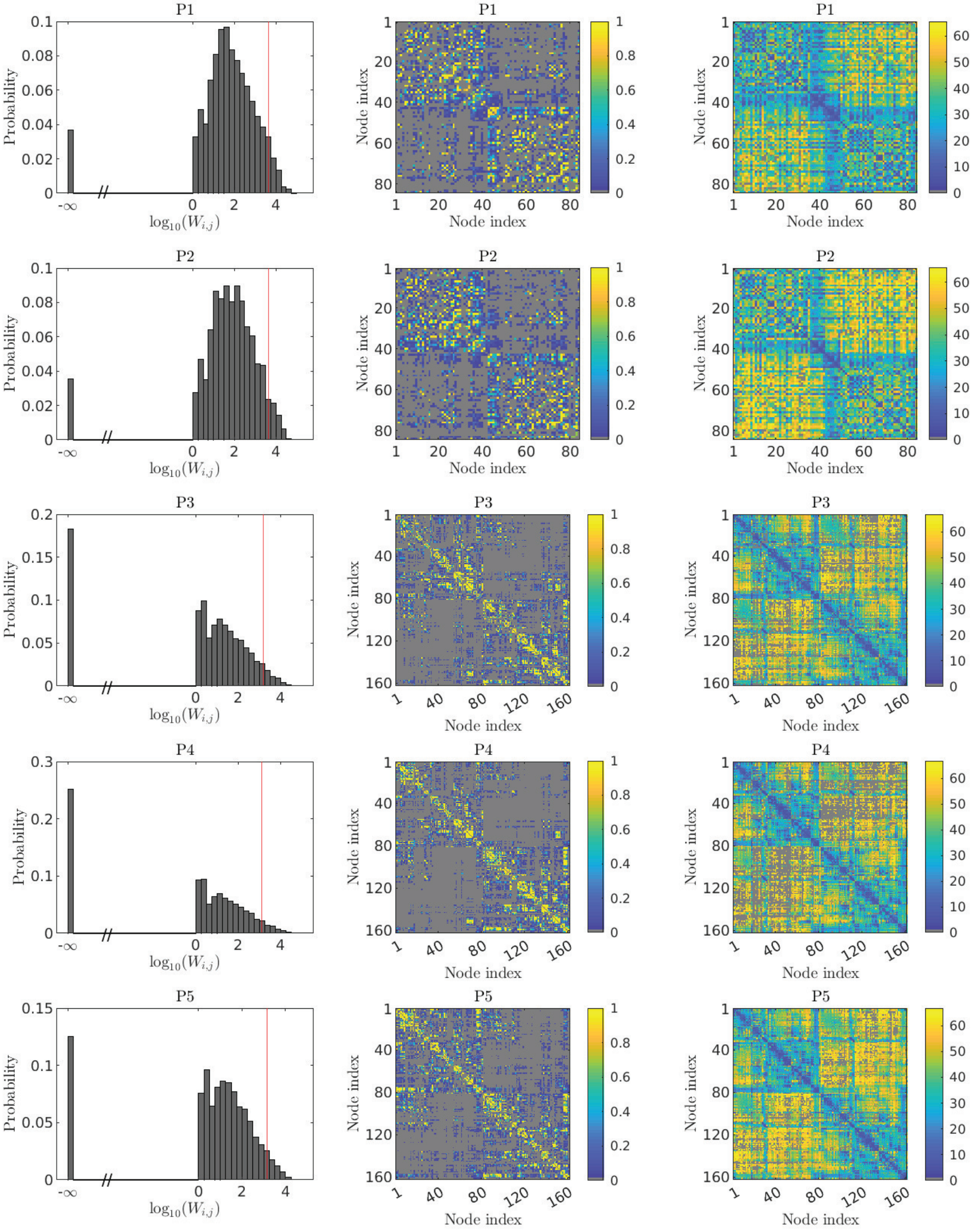

Supplementary Figure 1: **Left** distribution of patient-specific (non-normalized) connectivity weights. (From top to bottom: P1 – P5). The red line indicates the 95% percentile used as a threshold to obtain the truncated and normalized connectivity matrix. **Middle** normalized patient-specific connectivity matrices **W**. **Right** Corresponding propagation time delays τ_{ij} (in milliseconds).

Network	Clustering coefficient	Mean shortest path	Radius	Diameter	Small-world-ness
P1	0.9832	3.3645	5.5939	9.8978	1.6228
P2	0.9834	3.2260	4.6086	8.7336	1.5253
P3	0.9149	2.8358	3.6425	6.6711	1.3248
P4	0.8918	3.0257	4.1177	6.6789	1.3476
P5	0.9375	2.9441	4.0000	6.2852	1.4215

Table 1: Graph theoretic measures

In the above table, we used graph theoretic measures defined for weighted networks. For calculation of the shortest path length, the diameter and the radius of the network, the inverse of weights ($L_{i,j} = 1/W_{ij}$) were used. In this way, the shortest path length between two nodes is the minimum sum of the L_{ij} between the two nodes over all the possible paths. Diameter is the maximum of longest paths between all the pairs. Radius is defined as the minimum of the longest paths. The clustering coefficient measures how strongly the nearest neighbors of a node are connected to each other and is calculated as

$$C_i = \frac{1}{(k_i - 1)\sum_j W_{ij}} \sum_{j,k} \frac{W_{ij} + W_{ik}}{2} a_{ij} a_{ik} a_{jk} \tag{1}$$

where $a_{ij} = 1$ if there is a connection from j to i and zero otherwise. Small-world-ness is defined as

$$SWS = \frac{C_R L}{C L_R} \tag{2}$$

where C and L are the average clustering coefficient and the average shortest path length of the network, respectively. C_R and L_R are the corresponding measures obtained from random networks with the same size and weight distribution of as the patient-specific networks used in this study.

Phase transition diagrams and prediction based on local linear stability analysis

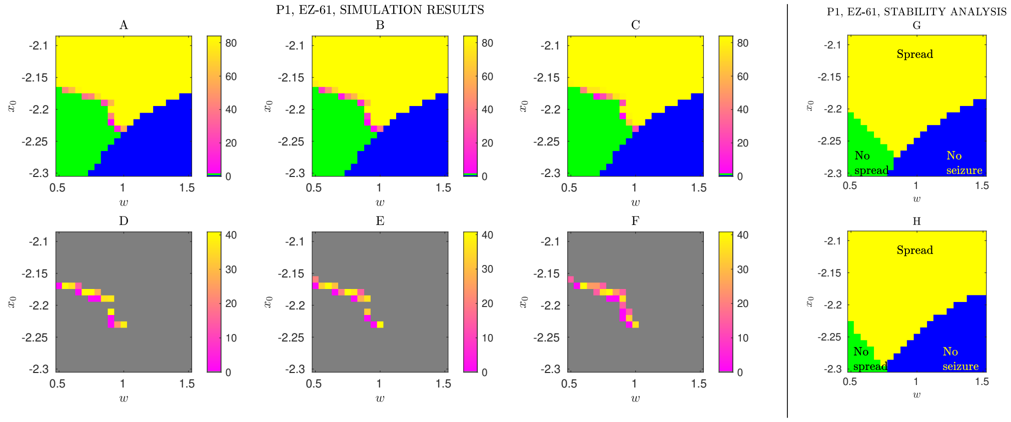

Supplementary Figure 2: P1, active EZ node 61. Same conventions as in Fig. 2, main text.

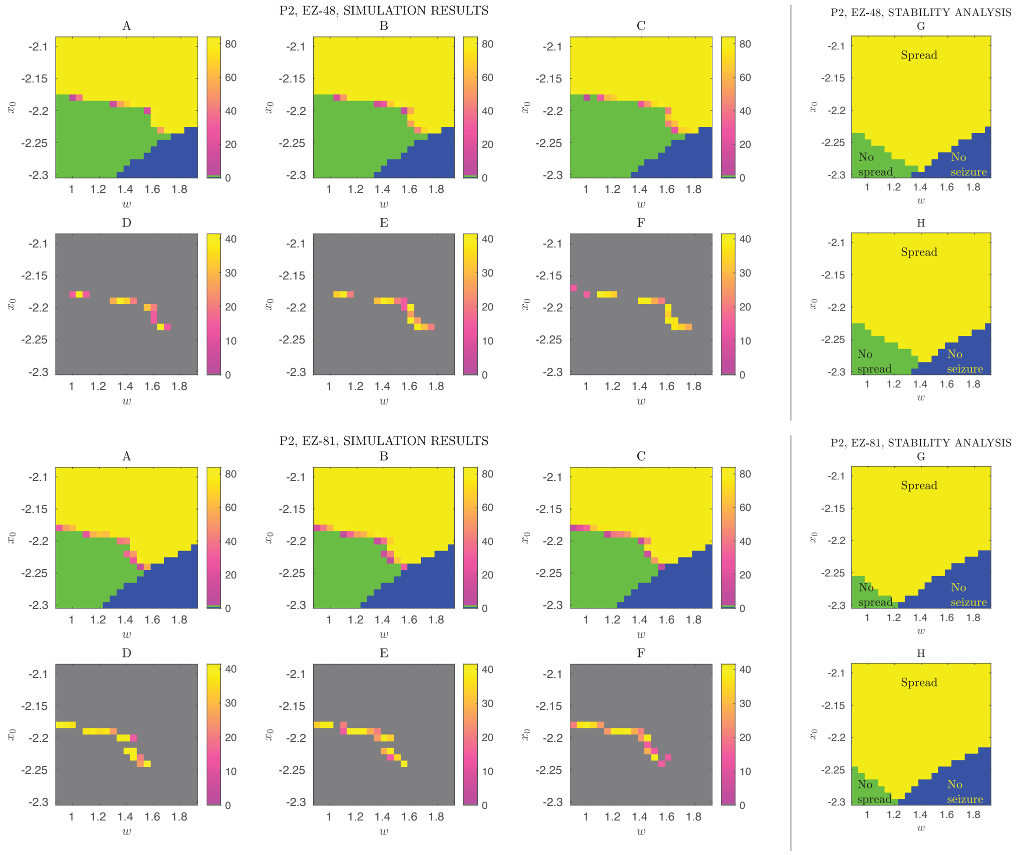

Supplementary Figure 3: P2. **Top A-H:** node 48 as the active EZ. **Bottom A-H:** node 81 as the active EZ.

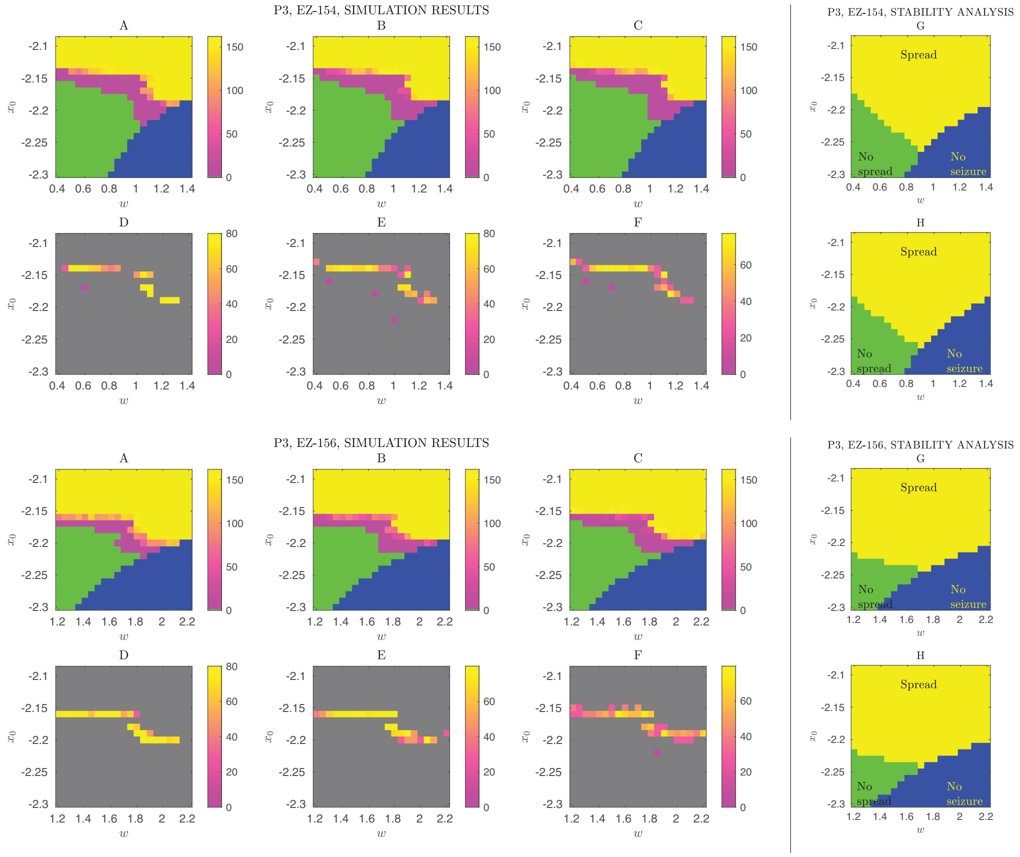

Supplementary Figure 4: P3. **Top A-H:** node 154 as the active EZ. **Bottom A-H:** node 156 as the active EZ.

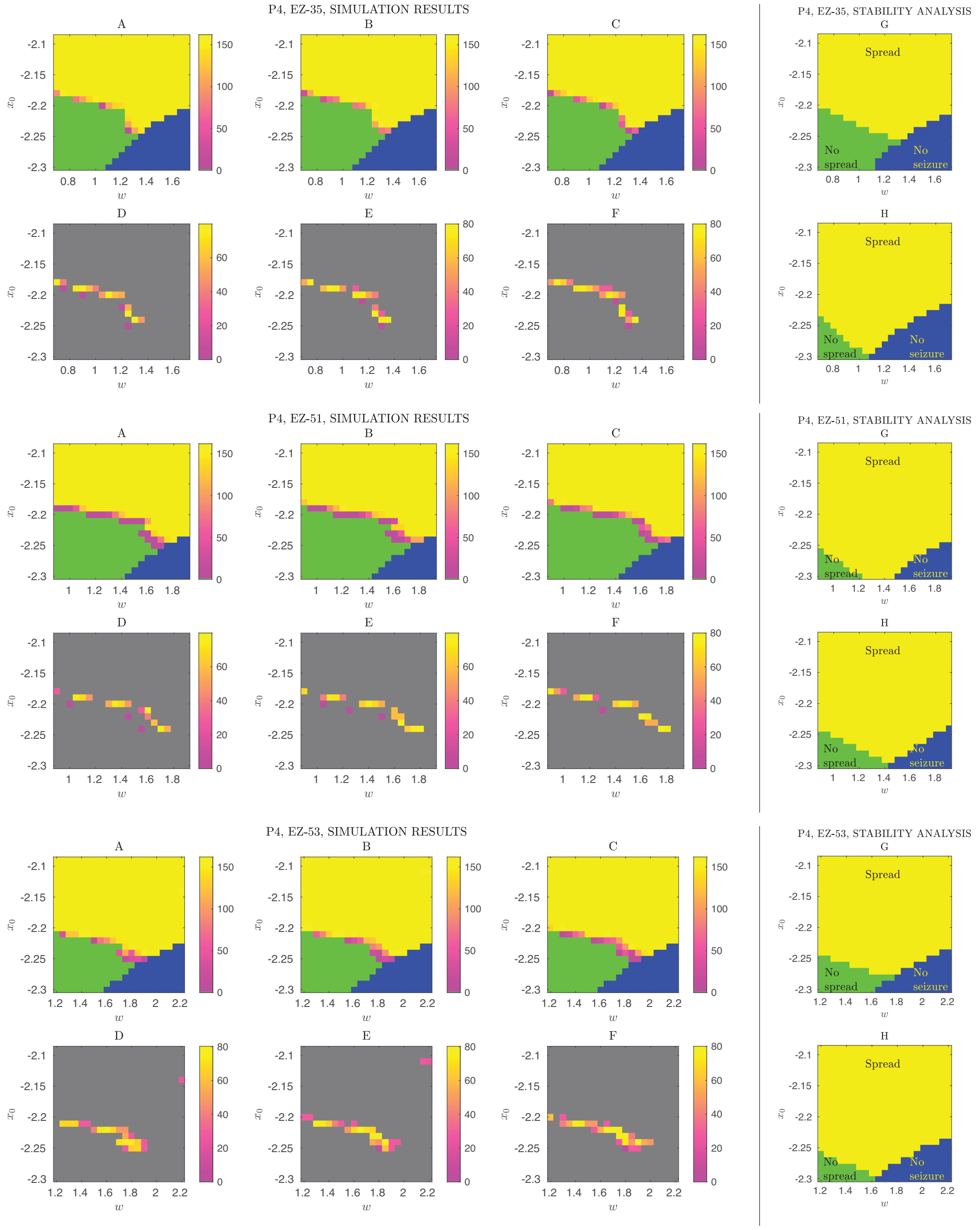

Supplementary Figure 5: P4. From top to bottom (A-H), nodes 35, 51, 53 as the active EZs, respectively.

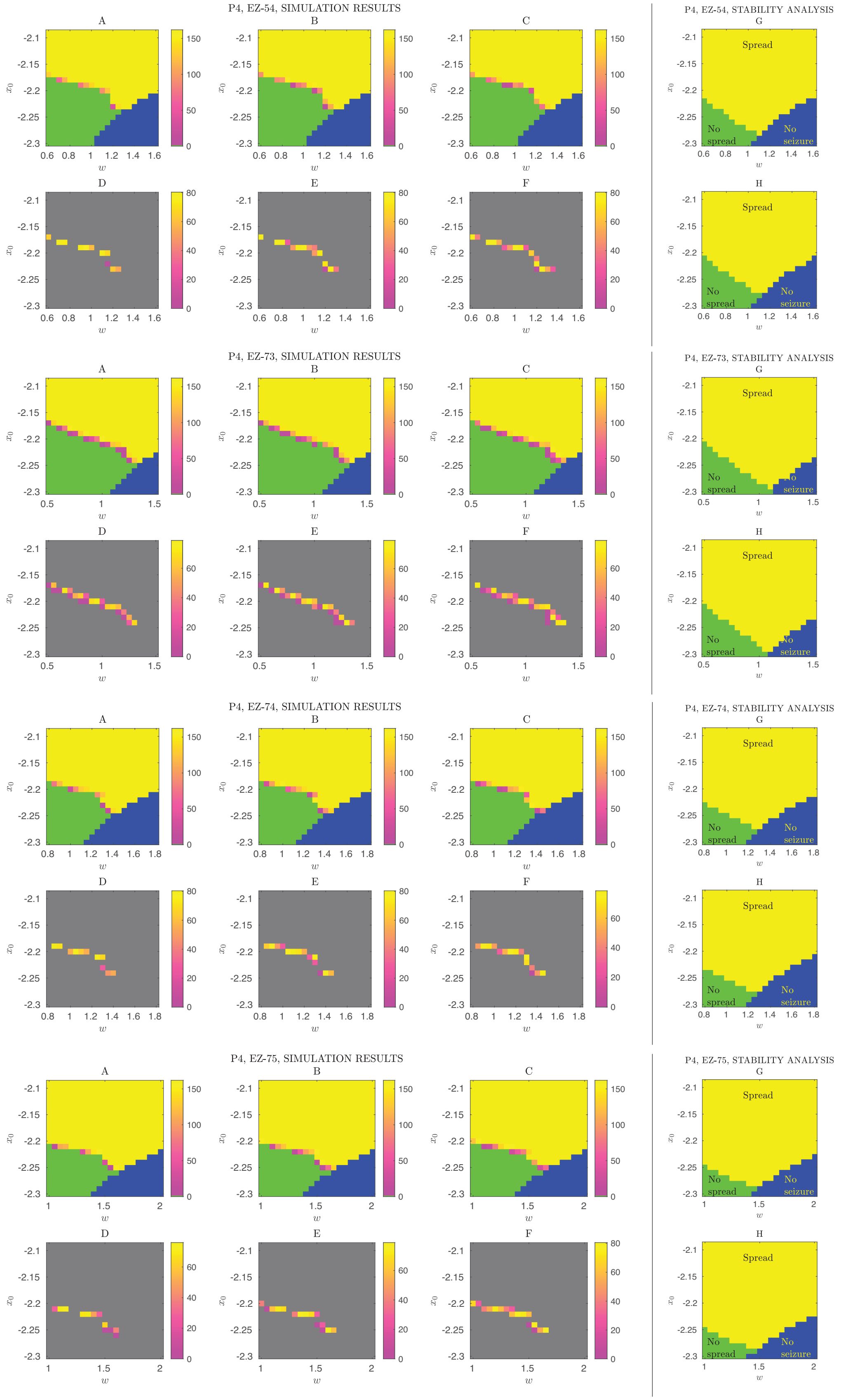

Supplementary Figure 6: P4. From top to bottom (A-H), nodes 54, 73, 74, 75 as the active EZs, respectively.

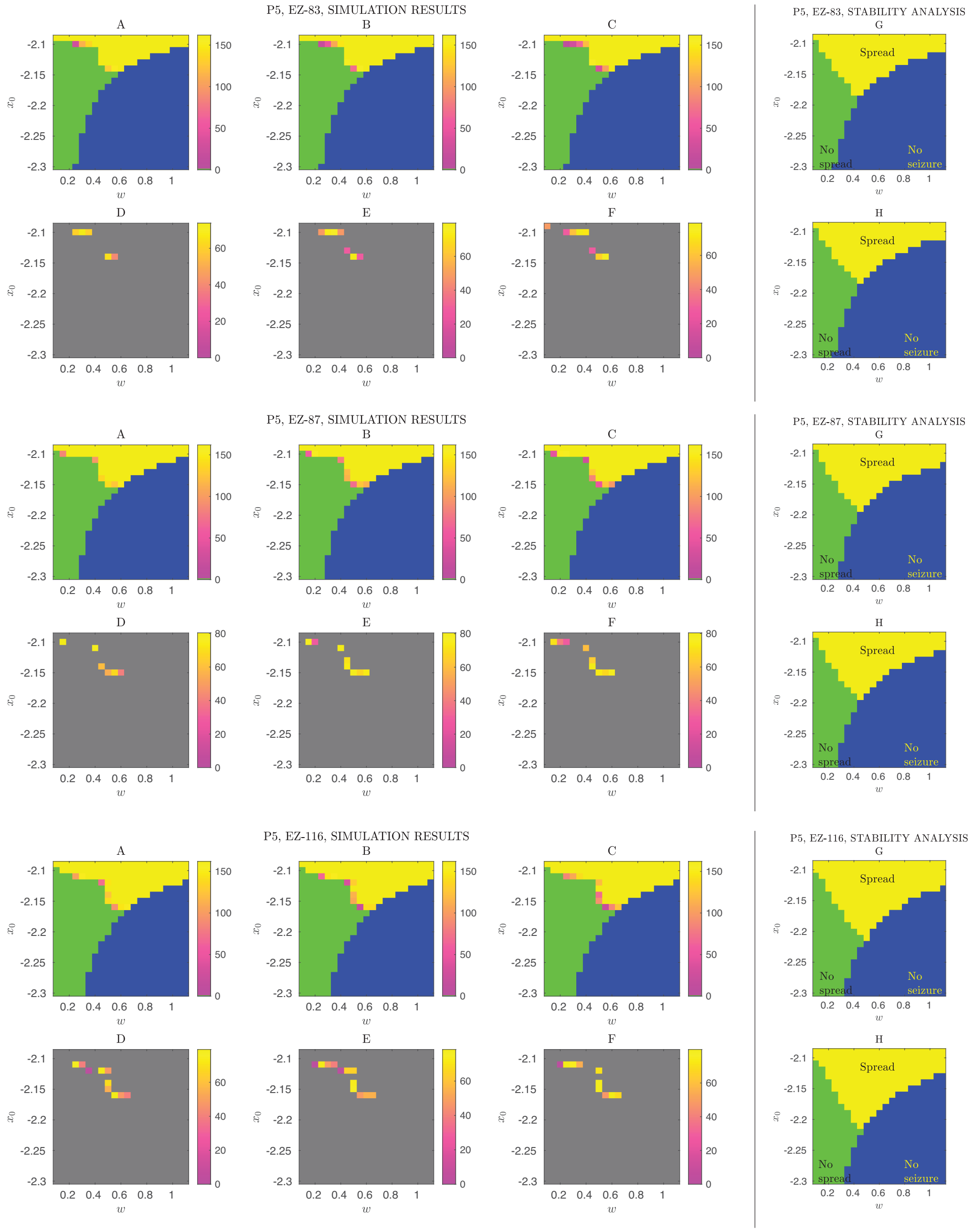

Supplementary Figure 7: P5. From top to bottom (A-H), nodes $83, 87, 116$ as the active EZs, respectively.

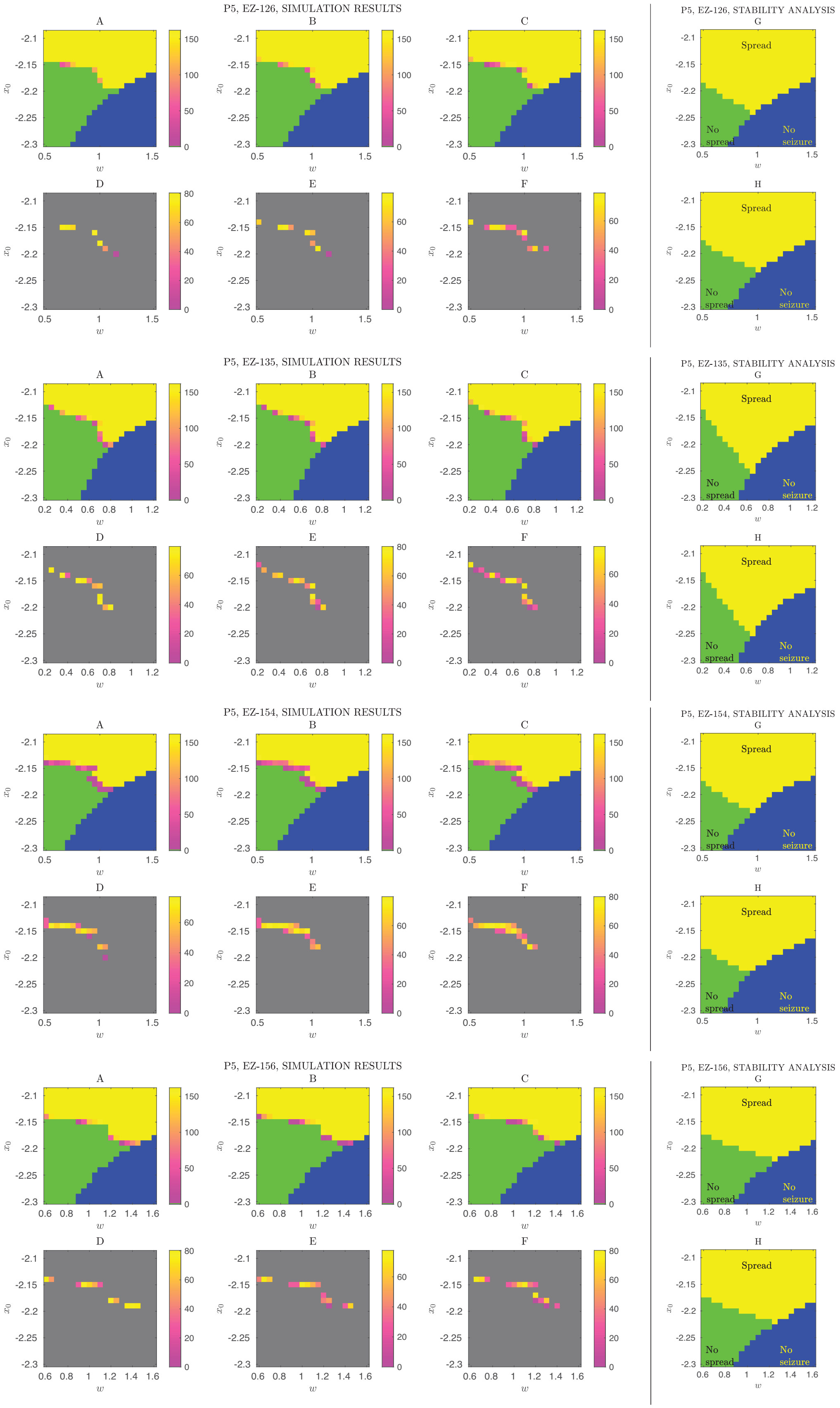

Supplementary Figure 8: P5. From top to bottom (A-H), nodes $126, 135, 154, 156$ as the active EZs, respectively.

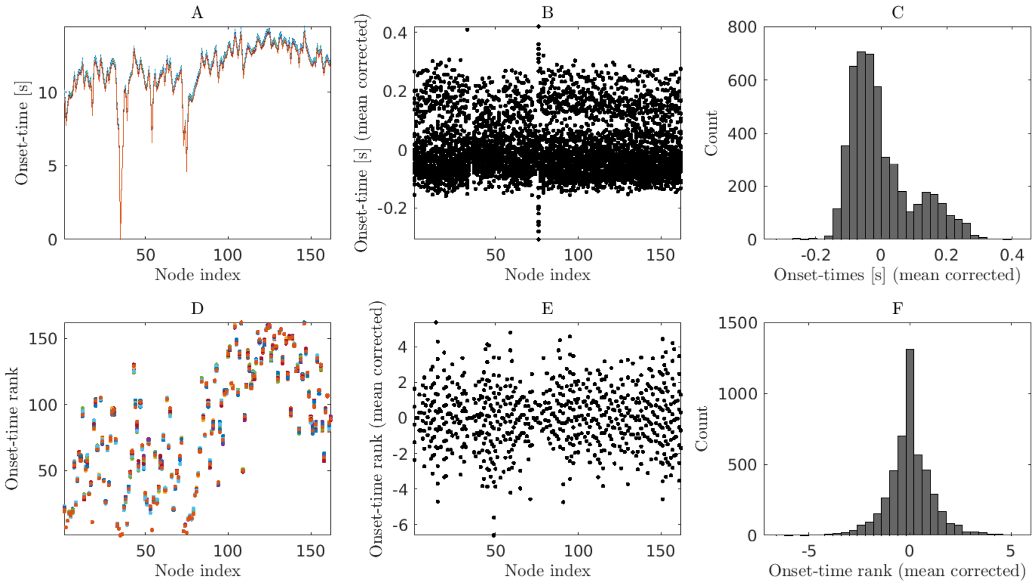

Supplementary Figure 9: Spread path variability across different stochastic realizations – Example from P4, EZ: node 35. (**A**) 30 realizations of seizure spread with different random seeds, under fixed parameters for surround excitability and global connectivity strength set to $x_0 = -2.13$ and $w = 1.5$, respectively. Onset times are centered at EZ seizure onset-time. (**B**) The data shown in **A** with mean subtracted for each node. (**C**) Histogram of mean corrected seizure onset-times across all nodes and realizations. (**D**) Same as **A** but for onset time ranking (lower rank numbers are associated with earlier onset times). The visible separation in two blocks reflects the two-hemisphere modular organization of the brain network and EZ location. (**E**) Mean-subtracted onset time ranks. (**F**) Histogram of spread rank across all nodes and realizations.

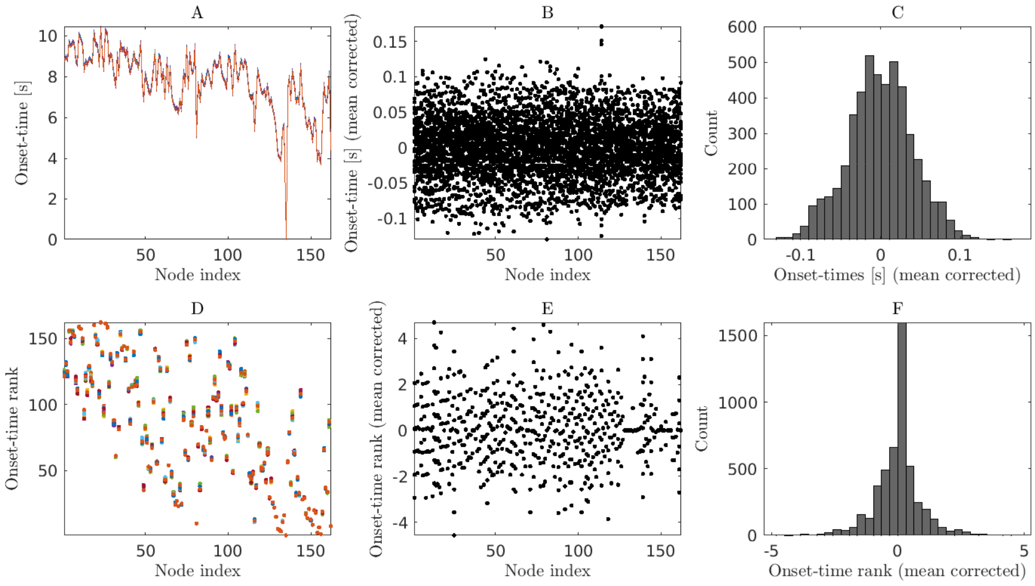

Supplementary Figure 10: Spread path variability among different stochastic realizations – Example from P3, EZ: node 135. Based on 30 realizations of seizure spread with different random seeds, under fixed parameters for surround excitability and global connectivity strength set to $x_0 = -2.13$ and $w = 1.1$, respectively. Same conventions as in Supplementary Figure 9.

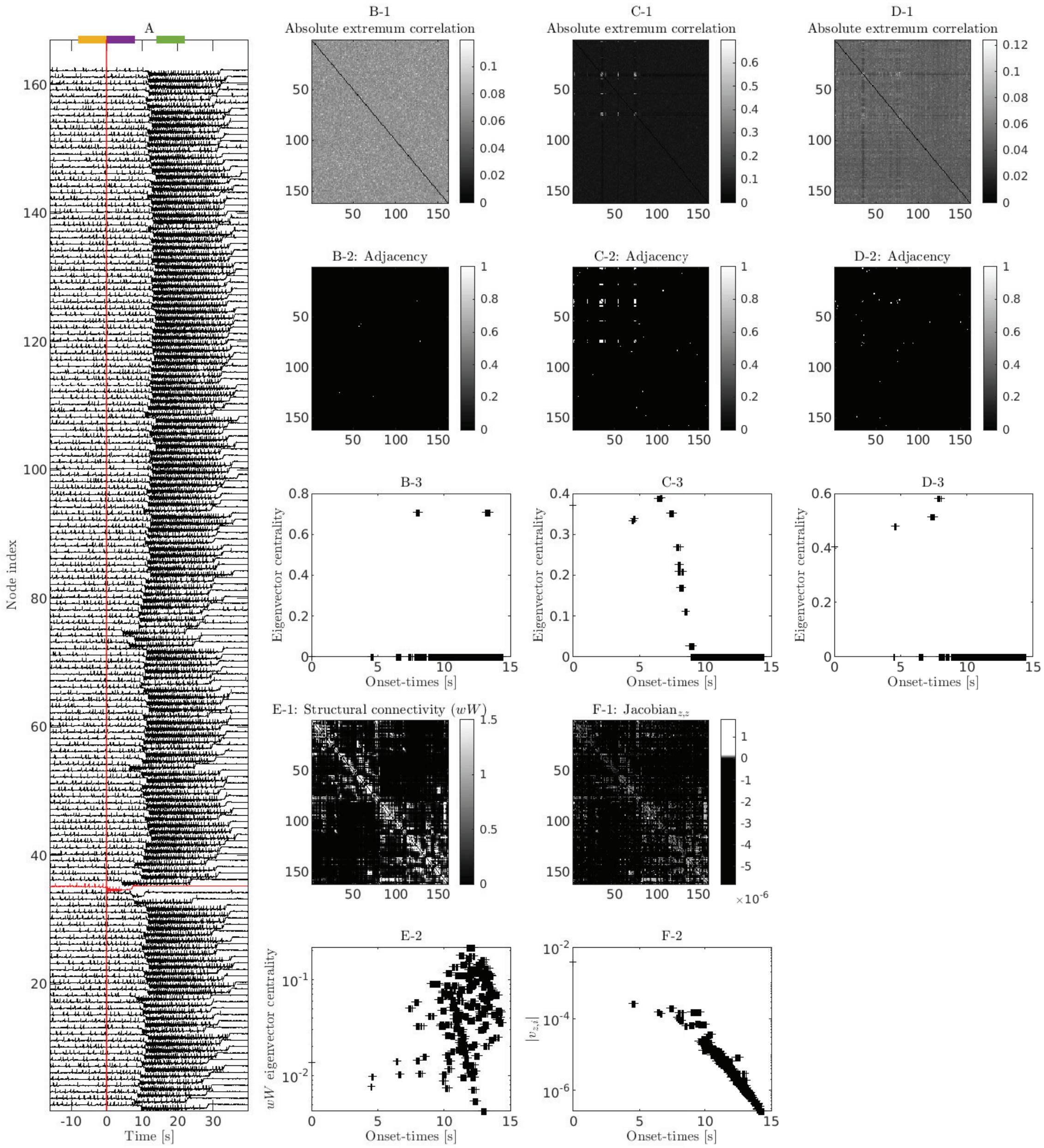

Supplementary Figure 11: Eigenvector centrality does not predict surround seizure onset-times in the epileptor network model – Example from P4, EZ: node 35. (**A**) A single realization of seizure spread in this patient-specific epileptor network, with surround excitability and global connectivity strength set to $x_0 = -2.13$ and $w = 1.5$, respectively. The EZ node activity is shown in red. Three 8-second long time windows at different time locations with respect to the seizure onset in the EZ are indicated by the bars with different colors at the top: 1) just before seizure onset in EZ node (orange); 2) just after seizure initiation (purple), and 3) during the full seizure (green). Time series from these 3 different windows, including data from 30 stochastic realizations, were used to estimate functional connectivity matrices. We used the algorithms and codes from Kramer et al., 2009. (**B-1**) Absolute extrema of cross-correlations functions between pairs of nodes for the time window before seizure initiation. (**B-2**) The corresponding (binary) adjacency matrix obtained by setting statistically significant absolute extrema to 1 ($p < 0.05$). (*Next page continuation ...*)

Supplementary Figure 11 (*Continuation*): (**B-3**) Values of the eigenvector centrality's components versus the seizure onset-times for the corresponding surround node (each data point corresponds to a node and a different stochastic realization).(**C-1,2,3**) and (**D-1,2,3**) same conventions as in **B-1,2,3**, but for the second and third time windows. (**E-1**) Patient-specific structural connectivity matrix W (white-matter tractography) scaled by the global connectivity strength parameter w. (**E-2**) Values of the corresponding eigenvector centrality's components versus the seizure onset-times for the corresponding surround node. (**F-1**) The Jacobian matrix with entries corresponding only to the permittivity variable z in the epileptor network model. (**F-2**) Values of the leading eigenvector's z components versus the seizure onset-times for the corresponding surround node. (The leading eigenvector was computed from the full Jacobian matrix.) As can be seen, the good predictability of seizure onset-times based on the leading eigenvector of the Jacobian matrix is not achieved when using eigenvector centrality computed either from adjacency matrices (functional connectivity) or from the structural connectivity matrix.

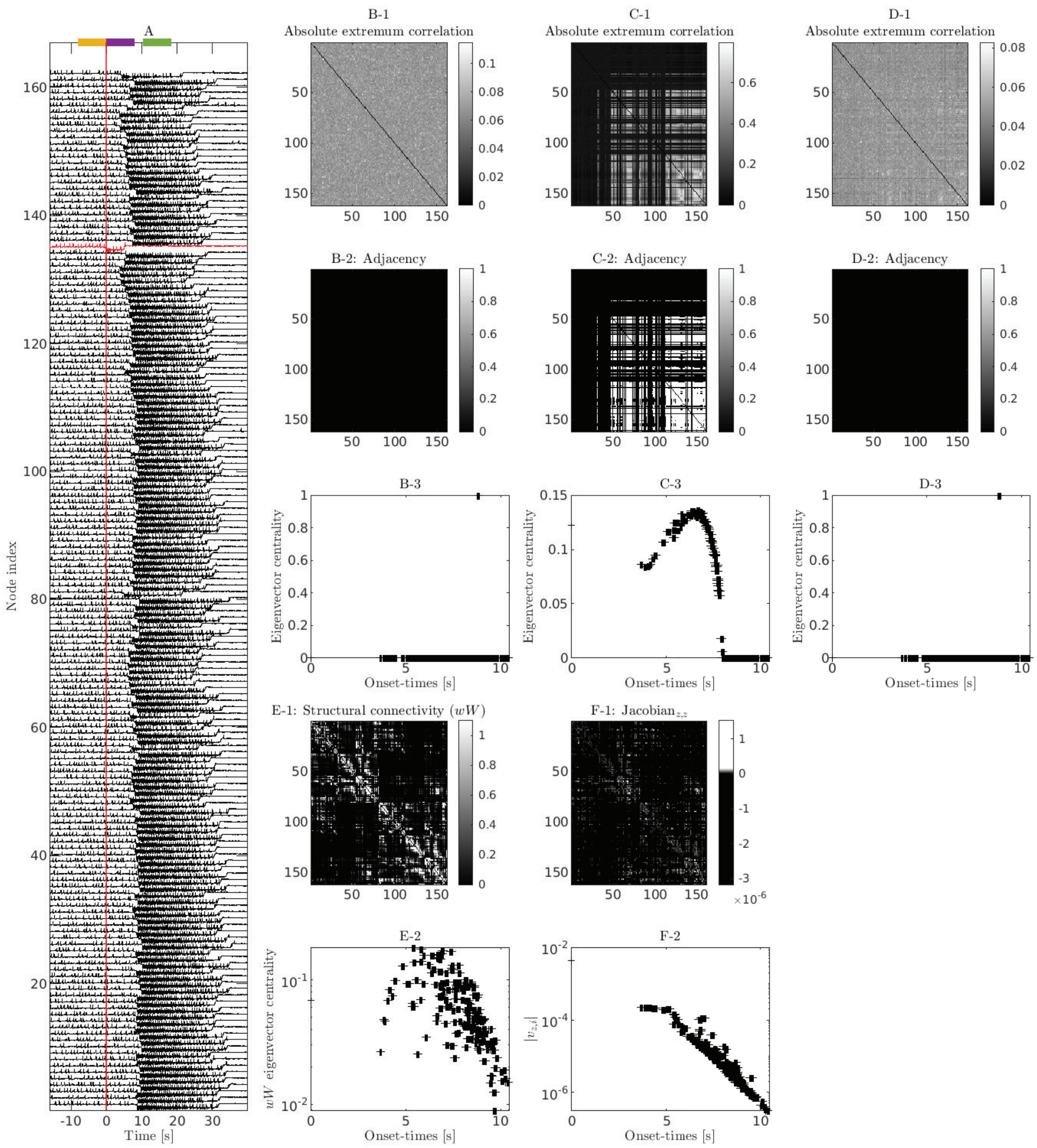

Supplementary Figure 12: Eigenvector centrality does not predict surround seizure onset-times in the epileptor network model – Example from P3, EZ: node 135 . Same conventions as in Supplementary Figure 11, but with surround excitability and global connectivity strength set to $x_0 = -2.13$ and $w = 1.1$, respectively.
